# Coral mucus rapidly induces chemokinesis and genome-wide transcriptional shifts toward early pathogenesis in a bacterial coral pathogen

**DOI:** 10.1038/s41396-021-01024-7

**Published:** 2021-06-24

**Authors:** Cherry Gao, Melissa Garren, Kevin Penn, Vicente I. Fernandez, Justin R. Seymour, Janelle R. Thompson, Jean-Baptiste Raina, Roman Stocker

**Affiliations:** 1 Department of Biological Engineering, Massachusetts Institute of Technology, Cambridge, MA USA; 2 Department of Civil and Environmental Engineering, Ralph M. Parsons Laboratory, Massachusetts Institute of Technology, Cambridge, MA USA; 3 Department of Civil, Environmental and Geomatic Engineering, Institute of Environmental Engineering, ETH Zurich, Zurich, Switzerland; 4 Working Ocean Strategies LLC, Carmel, CA USA; 5 Department of Applied Environmental Science, California State University Monterey Bay, Seaside, CA USA; 6 Climate Change Cluster (C3), University of Technology Sydney, Ultimo, NSW Australia; 7 Singapore Center for Environmental Life Sciences Engineering, Nanyang Technological University, Singapore, Singapore; 8 Asian School of the Environment, Nanyang Technological University, Singapore, Singapore

**Keywords:** Microbial ecology, Microbial ecology, Marine microbiology, Bacterial pathogenesis

## Abstract

Elevated seawater temperatures have contributed to the rise of coral disease mediated by bacterial pathogens, such as the globally distributed *Vibrio coralliilyticus*, which utilizes coral mucus as a chemical cue to locate stressed corals. However, the physiological events in the pathogens that follow their entry into the coral host environment remain unknown. Here, we present simultaneous measurements of the behavioral and transcriptional responses of *V. coralliilyticus* BAA-450 incubated in coral mucus. Video microscopy revealed a strong and rapid chemokinetic behavioral response by the pathogen, characterized by a two-fold increase in average swimming speed within 6 min of coral mucus exposure. RNA sequencing showed that this bacterial behavior was accompanied by an equally rapid differential expression of 53% of the genes in the *V. coralliilyticus* genome. Specifically, transcript abundance 10 min after mucus exposure showed upregulation of genes involved in quorum sensing, biofilm formation, and nutrient metabolism, and downregulation of flagella synthesis and chemotaxis genes. After 60 min, we observed upregulation of genes associated with virulence, including zinc metalloproteases responsible for causing coral tissue damage and algal symbiont photoinactivation, and secretion systems that may export toxins. Together, our results suggest that *V. coralliilyticus* employs a suite of behavioral and transcriptional responses to rapidly shift into a distinct infection mode within minutes of exposure to the coral microenvironment.

## Introduction

Coral reefs are declining worldwide due to rising sea surface temperatures and increasing prevalence of coral disease outbreaks [1–3]. Elevated sea surface temperatures cause physiological stress in corals [4] and provide distinct advantages for some coral pathogens [5]. One well-studied coral pathogen, *Vibrio coralliilyticus* BAA-450, displays tightly regulated temperature-dependent virulence against its coral host, *Pocillopora damicornis*. While this *V. coralliilyticus* strain is avirulent at temperatures below 24 °C, it is capable of attacking the coral symbiotic dinoflagellates [6, 7] and lysing coral tissue [8] at temperatures above 27 °C.

*V. coralliilyticus* displays two distinct behavioral adaptations enabling targeted infection of corals that are physiologically stressed and therefore more vulnerable to pathogenic invasion. First, the bacterial pathogen uses chemotaxis to target chemical signatures present in the mucus of stressed corals [9]. Second, *V. coralliilyticus* displays chemokinesis, which is the ability to change swimming speed in response to a change in chemical concentration, to potentially enable faster environmental exploration in the presence of its coral host mucus [9, 10]. While both chemotaxis and chemokinesis are behaviors associated with motility and chemical sensing, chemotaxis specifically refers to the ability to detect and follow chemical gradients (without necessarily any change in swimming speed), whereas chemokinesis refers to the ability to change swimming speed in response to an overall change in concentration in the environment (without any reference to whether cells follow gradients). In *V. coralliilyticus*, these two behaviors combine to enable efficient and rapid targeting of stressed corals. However, chemokinesis, in contrast to chemotaxis, has remained more rarely studied in bacteria and is almost entirely undescribed in the context of marine disease [11–13].

Coral mucus—in addition to triggering increased motility and chemotaxis which are behaviors necessary for infection by *V. coralliilyticus* [14, 15]—also represents the critical interface where pathogen activities can dictate the outcome of an infection. Corals secrete up to half of the carbon assimilated by their algal symbionts as mucus [16, 17] and its production represents a sizable energetic investment that is important for nutrient cycling across the entire reef system [18–21]. In addition, mucus provides corals with protection against desiccation and is an ancient and evolutionarily conserved first line of defense against pathogens [22]. During infection studies, corals have been observed to actively expel ingested pathogens by spewing out bacteria-laden mucus from the mouths of polyps [23, 24]. However, entry into host mucus may also signal to the bacterial pathogen that contact with a potential host is imminent. Thus, elucidating the behavioral and transcriptional responses of *V. coralliilyticus* in the context of its coral host environment, and in particular coral mucus, is important in elucidating the mechanisms underpinning coral infection.

Here, we present experiments in which we simultaneously used video microscopy and RNA sequencing to measure the behavioral and transcriptional responses of *V. coralliilyticus* upon a sudden exposure to mucus from its coral host. To study chemokinesis independently from chemotaxis, we conducted our experiments in the absence of chemical gradients. This represents the first investigation to couple behavioral and transcriptomic analyses to decipher the mechanisms promoting coral infection. We show that behavioral and transcriptional responses occur concomitantly over a surprisingly rapid timescale of only minutes, highlighting the agility of the pathogens in seizing what are likely to be limited windows of opportunity [25] to target and ultimately infect their host.

## Materials and methods

### Coral mucus collection

Five small colonies of the coral *Pocillopora damicornis* were collected from Heron Island (23.4423 °S, 151.9148 °E, Great Barrier Reef, Australia) in April 2015 and maintained in aquaria for 2 weeks at 25 °C ± 1 °C and 35 ppt salinity on a 12 h light–dark cycle. Mucus was collected with a sterile 1-ml syringe (Becton Dickinson, NJ, USA) from corals subjected to repeated 3-min air exposure, and snap-frozen in a sterile 50 ml tube (Falcon, Corning Life Sciences, MA, USA) in liquid nitrogen and maintained at −80 °C until use in experiments. Due to the large volume required (55 ml), mucus was collected and pooled from the five coral colonies over 3 consecutive days (separate snap-frozen tube per day). Directly before the experiments, a single pool of coral mucus was created by thawing in a room temperature water bath and pooling all samples into a sterile glass bottle.

### Bacterial culture

*Vibrio coralliilyticus* type strain ATCC BAA-450 was acquired from the American Type Culture Collection (Manassas, VA, USA). A frozen stock (−80 °C, 25% glycerol) of *V. coralliilyticus* was streaked onto a marine broth 2216 (BD Difco, Franklin Lakes, NJ, USA) culture plate and incubated at room temperature for 24–48 h. For liquid culture inoculation, five colonies were resuspended in 20 µl filtered (0.2 µm) artificial seawater (FASW, 35 g/L sea salt; Instant Ocean, Blacksburg, VA, USA) and 5 µl of this suspension was inoculated into each of three sterile 250-ml Erlenmeyer flasks containing 80 ml 1% marine broth medium (99% FASW, v/v). The timing of inoculation of the triplicate liquid cultures was staggered to allow 17–18 h of growth (to OD_600_ 0.04) before the start of each replicate experiment. Liquid cultures were incubated at 30 °C in an orbital shaker (250 rpm).

### Preparation of filtered spent medium

Experimental controls consisted of *V. coralliilyticus* cells incubated in their own filtered spent medium, which was prepared immediately prior (≤10 min) to the start of each replicate experiment. Approximately 20 ml *V. coralliilyticus* overnight culture was aseptically filtered through a sterile 0.2-µm syringe filter (polyethersulfone membrane; VWR International) attached to a sterile 10-ml syringe, into a sterile 50-ml Falcon tube. Filters were changed every 10 ml of volume filtered to minimize clogging.

### Experimental setup

**Fig. 1:**
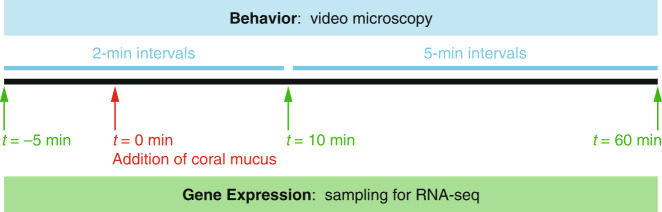
Behavioral and transcriptional measurements of *V. coralliilyticus* in coral mucus. Experimental timeline showing time points for microscopy video acquisition and RNA-seq sampling. At *t* = 0 min (red arrow), coral mucus (treatment) or filtered spent medium (control) was added (1:1, v/v) to *V. coralliilyticus* cultured in 1% marine broth. The experiment was conducted at 30 °C, and repeated three times sequentially in a single day, using three different cultures of *V. coralliilyticus*.

Three biological replicate experiments were sequentially performed on the same day using three independent cultures of *V. coralliilyticus* exposed to their respective filtered spent media (control) or aliquots of the same pooled coral mucus (treatment) (Fig. 1). Aliquots of 25 ml of bacteria were placed into each of two RNase-free 50-ml Falcon tubes for the mucus and control treatments. Bacteria (before and after addition of mucus), coral mucus, and filtered spent media were maintained at 30 °C ± 1 °C in a water bath for the duration of the experiment to prevent temperature shock. All experiments were performed at 30 °C to capture conditions in which *V. coralliilyticus* is capable of infecting its coral host [8]. Incubation was initiated by the 1:1 (v/v at *t* = 0 min) addition of coral mucus or filtered spent medium to bacteria, directly followed by thorough mixing by repeated pipetting to eliminate any chemical gradients.

Bacteria were repeatedly and manually sampled from populations incubated with mucus or filtered spent media. At each time point, 40 µl of the bacteria-containing solution was removed from the top of the incubation tube using a pipette and introduced into a straight microfluidic channel (60 mm × 4 mm, 90 µm depth), followed immediately by video acquisition. A single microfluidic device, fabricated using soft lithography [26] and permanently bonded to a glass microscope slide (75 mm × 25 mm × 1 mm; VWR International), contained two identical channels in parallel to accommodate the mucus and the control experimental conditions. A temperature-controlled microscope stage insert (Warner Instruments, Hamden, CT, USA) was used to maintain cells at 30 °C ± 1 °C during imaging. After each time point, cells were removed from the device and discarded using a pipette, before the introduction of freshly sampled cells at the next time point. A new microfluidic device was used for each replicate experiment.

### Microscopy and video analysis

Experiments were imaged using phase-contrast microscopy on an inverted epifluorescence TE2000 microscope (Nikon, Tokyo, Japan), controlled through Nikon Elements software, with a 20× objective (S Plan Fluor ELWD ADM Ph1 20×, 0.45 NA; Nikon) and 1.5× optical magnification (combined objective magnification of 30×). An sCMOS camera (Andor Neo, 2560 × 2160 pixels, 6.5 µm/pixel; Andor, Belfast, Northern Ireland) was used to acquire videos (300 frames at 29.79 fps) from the center of the microfluidic channels, with the focal plane at channel mid-depth to avoid wall effects on motility.

**Fig. 2:**
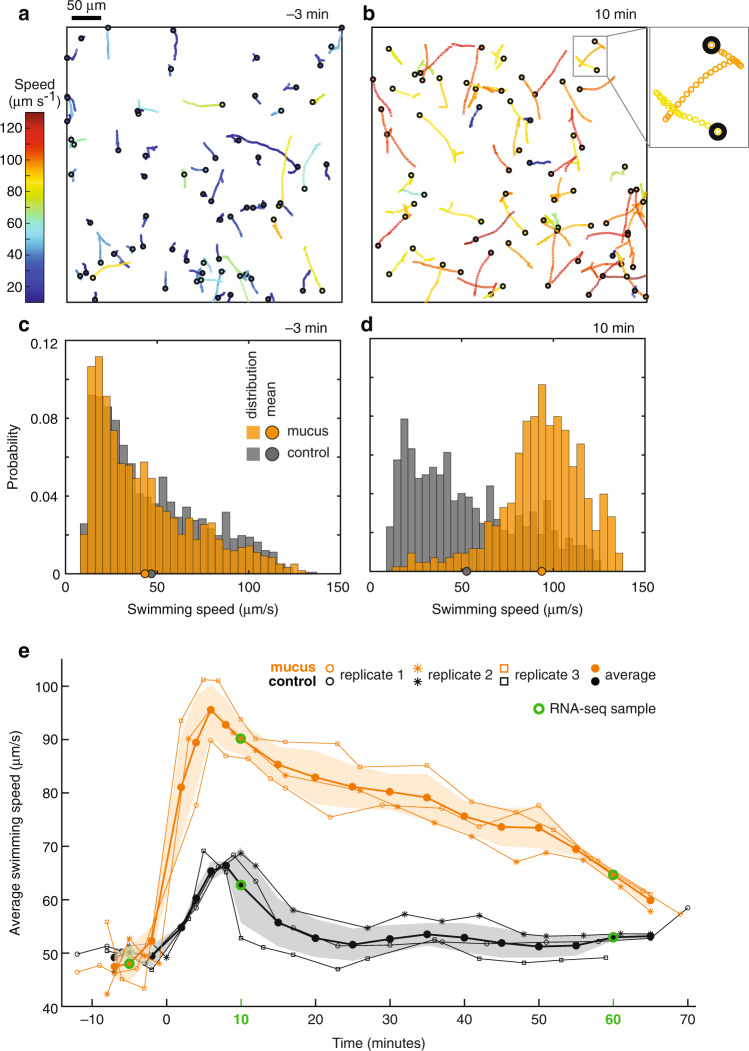
*V. coralliilyticus* exhibits strong chemokinesis upon exposure to coral mucus. Experiments were conducted at 30 °C. **a**, **b** Swimming tracks of *V. coralliilyticus* before (−3 min, **a**) and after (10 min, **b**) addition of coral mucus. Swimming tracks of 70 motile cells were randomly selected for each panel. Black circles mark the start, and colors indicate the mean swimming speed, of each track. Colored circles (3× zoom of gray box provided for visibility) represent frames of a microscopy video (0.03 s between frames). **c**, **d** Probability distributions of swimming speeds of motile cells, before (−3 min, **c**) and after (10 min, **d**) addition of coral mucus (orange) or filtered spent medium (gray). **e** Average swimming speeds of motile cells before and after 1:1 addition (v/v at *t* = 0 min) of coral mucus (orange) or filtered spent medium (black, control). Averages (filled circles) and standard deviation (shaded regions) were calculated using data obtained from three replicate experiments (○, *, □). Data were interpolated to match time points across replicates. RNA-seq samples were taken at −5, 10, and 60 min (green circles). Data from replicate 3 are presented in panels (**a**–**d**).

Analysis of microscopy videos was performed in MATLAB (MathWorks, Natick, MA, USA) using an automated image segmentation and trajectory reconstruction software developed in-house (detailed methods in Supplementary Methods). Briefly, cells in each frame were identified using a pixel intensity threshold, and their swimming trajectories were reconstructed using their *x, y*-positions in sequential frames (Fig. 2a, b). To discriminate motile from non-motile bacteria, we determined the type of motion (ballistic or diffusive) of each bacterium by calculating the mean squared displacement (MSD) as a function of short time intervals (Δ*t)*, and quantifying the exponent α of this dependence (MSD ~ Δ*t*^α^). Non-motile bacteria were identified as slow-moving cells (median instantaneous speed < 10 µm/s) displaying purely diffusive motion (*α* < 1). Motile bacteria were instead identified as those cells moving more rapidly (median instantaneous speed ≥ 10 µm/s) and having a higher value of the MSD exponent (*α* ≥ 1) (Supplementary Fig. 1). A sensitivity analysis showed that our results were robust against the selection of different threshold values (Supplementary Fig. 2). Amongst motile bacteria, the swimming speed of each cell was calculated by averaging the instantaneous speed over the duration of its trajectory, and the mean speed of the population was quantified by averaging over all trajectories of motile bacteria detected in each microscopy video (mean ± s.d. of number of trajectories in videos, *n* = 1298 ± 312 pre-addition, *n* = 753 ± 230 post-addition; Supplementary Fig. 3), which represented a single time point in mucus or control conditions (Fig. 2).

### RNA sampling, isolation, sequencing, and sequence alignment

To characterize changes in gene expression accompanying shifts in swimming behavior, RNA sequencing (RNA-seq) was performed on the same *V. coralliilyticus* populations from which samples for tracking by video microscopy were obtained. Incubation tubes containing bacteria were swirled vigorously to mix before taking samples of mucus and control cells at three time points during each replicate experiment (Fig. 1). Sample volumes of 8 ml before mucus or filtered spent medium (control) addition (*t* = 0 min), and 16 ml after addition (*t* = 10 min and  *t* = 60 min; larger volume to account for biomass dilution) were each filtered through a 0.22-µm Sterivex-GP filter unit (polyethersulfone membrane; Millipore) attached to a sterile 60-ml syringe to capture bacteria onto the membrane. Subsequently, the filtrate end of the Sterivex filter cartridge was briefly flamed and pinched to close, and 2 ml of RNAlater (Thermo Fisher Scientific, Waltham, MA, USA) was added with a pipette through the other end of the cartridge to immerse the cell-containing filter membrane in the RNA-stabilizing solution. Sample-containing filter cartridges were incubated at 4 °C for 24 h, then preserved at −80 °C until RNA extraction. A sample of coral mucus (~2 ml) was also preserved, and its RNA extraction confirmed that little to no RNA was present in coral mucus.

Total RNA extraction was performed as described previously [27] (detailed description in Supplementary Methods). Depletion of rRNA (using Ribo-Zero rRNA Removal Kit for bacteria (Epicentre Biotechnologies)) and subsequent mRNA sequencing (using an Illumina HiSeq2500 sequencer), assembly, and alignment were performed by the Joint Genome Institute (Los Alamos, NM, USA) (detailed description in Supplementary Methods). Due to RNA degradation, the mucus-treatment sample at 10 min from replicate 2 could not be sequenced. Thus, 17 samples in total were sequenced (Sequence Read Archive accession PRJNA707316). Raw reads from each library were aligned to the reference genome (*V. coralliilyticus* ATCC BAA-450, NCBI Taxon ID 675814). As a result, 99.48% (5022 genes) of the filtered FASTQ reads mapped to the *V. coralliilyticus* reference genome. GenBank protein IDs (prefix “EEX”) were obtained from *V. coralliilyticus* BAA-450 genome assembly ASM17613v1 and matched by locus tags (prefix “VIC_”) of each gene.

### Differential expression analysis

The DESeq2 package [28] (v1.26.0) in R was used for differential expression analyses. The DESeq2 algorithm uses negative binomial generalized linear models to test for differential abundances in raw count data, and controls for differences in sequencing depth between libraries by estimating size and dispersion factors. Adjusted *p* values were calculated in DESeq2 using the Benjamini-Hochberg procedure, which accounts for multiple comparisons. Statistically significant gene expression differences were assessed using the Wald test with a false discovery rate (FDR) cutoff at adjusted *p* < 0.01. No fold-change cutoff was applied. Time-point-matched, pairwise mucus–control comparisons were performed. All instances of gene differential expression given in the text are statistically significant (unless otherwise noted), and the fold-difference values provided in each case are relative to the control at the same time point.

For principal component analysis (PCA) and sample-to-sample distance calculation, raw count data were transformed using the variance stabilizing transformation (VST) method [29] to remove the dependence of the variance on the count mean, especially when count means are low. VST uses the experiment-wide trend of variance over mean in order to transform the data to remove the experiment-wide trend. Transformed values are on the log_2_ scale.

### Gene set enrichment analysis (GSEA)

Gene set enrichment analysis was performed using the GSEA software [30, 31] v4.0.3 to identify differential representation of metabolic pathways in transcripts sequenced in mucus or control conditions. Only genes with significant differential expression determined by DESeq2 were included in the GSEA analyses (GSEAPreranked protocol). Gene sets were defined by KEGG PATHWAY, and significance was determined at an FDR *q* value cutoff of 0.25. Genes were ranked by log_2_ fold-change values. Further details are described in Supplementary Methods.

## Results

### *V. coralliilyticus* exhibits strong chemokinesis within minutes of exposure to coral mucus

Video microscopy revealed a strong and rapid behavioral response by *V. coralliilyticus* cells following exposure to *Pocillopora damicornis* coral mucus (Fig. 2) at 30 °C, a temperature at which this pathogen is capable of infecting its coral host [8]. The viscosity of coral mucus in our experiments (0.750 cP) was similar to that of filtered artificial seawater (0.731 cP) at room temperature (Supplementary Methods). Thus, bacterial responses measured in this study were assumed to be mostly due to chemical components of coral mucus, although temperature-dependent viscosity changes of coral mucus may influence bacterial swimming [32, 33].

*V. coralliilyticus* responded to coral mucus with strong chemokinesis. Video microscopy revealed that the average swimming speed of *V. coralliilyticus* cells increased from 48.0 ± 3.4 µm/s (mean ± s.d.), measured 5 min before addition of coral mucus, to 81.0 ± 9.6 µm/s within 2 min of coral mucus addition (the first time point at which speed was measured post mucus addition; Fig. 2e). The maximum swimming speed (95.5 ± 4.6 µm/s) was reached at 6 min post-addition, representing a two-fold increase compared to the pre-addition state (Fig. 2e). After reaching their maximum speed, the average swimming speed of bacteria in coral mucus gradually decreased over time, but remained significantly higher than the swimming speeds of pre-addition and control cells for the entire experimental duration (65 min; two-tailed *t* tests, *p* < 0.01). When samples were collected for RNA-seq, at 10- and 60-min post-addition of mucus, swimming speeds were 90.1 ± 2.9 µm/s and 64.5 ± 0.9 µm/s, or 1.9× or 1.3× compared to baseline, respectively (Fig. 2e).

Chemokinesis by *V. coralliilyticus* is a temperature-dependent response and appears to be driven by the influx of nutrients and other signaling molecules. First, coral mucus–induced chemokinesis was attenuated in an experiment conducted at a lower temperature (18.7 °C), in which the observed speed enhancement was only 1.1-fold at 7 min post mucus addition (Supplementary Fig. 4). Second, in a separate experiment conducted at 30 °C, rich medium and coral mucus both led to a doubling of swimming speed (2.3-fold increase for rich medium, 2.2-fold increase for coral mucus) within 5 min (Supplementary Fig. 5), suggesting that, at least partially, chemokinesis is a response to the nutrient influx upon addition of mucus. Third, control cells, exposed to filtered spent medium in place of coral mucus, displayed a weak and short-lived increase in swimming speed that peaked at 1.3-fold the pre-addition speed after 8 min and returned to baseline within 15 min (Fig. 2e), which may have been caused by oxygenation of the spent medium during filtration (Supplementary Discussion). Taken together, our results suggest that the swimming speed of *V. coralliilyticus* is highly sensitive to chemical changes in the environment, and that at the virulence-inducing temperature of 30 °C, chemokinesis in response to coral mucus is likely due to the influx of nutrients, and is especially rapid, larger in magnitude, and longer in duration compared to any other conditions tested.

Probability distributions of the swimming speeds of motile cells before and after mucus addition showed that the observed increase in average swimming speed was caused by a shift of the entire motile *V. coralliilyticus* population toward a faster swimming regime (Fig. 2c, d, Supplementary Fig. 6), rather than the emergence of behaviorally distinct subpopulations. In contrast, only a subset of motile control cells increased swimming speeds upon the addition of filtered spent media (Supplementary Fig. 6). Furthermore, ~75% of the population was motile over the whole experimental duration in both conditions (exposed to mucus or spent medium;  Supplementary Fig. 7). These results suggest that speed enhancement within the motile fraction of the population underlies the increase in average swimming speed observed in coral mucus, and that non-motile cells mostly maintain their non-swimming state upon mucus addition.

### Within minutes of coral mucus exposure, *V. coralliilyticus* initiates a transcriptional response

**Fig. 3:**
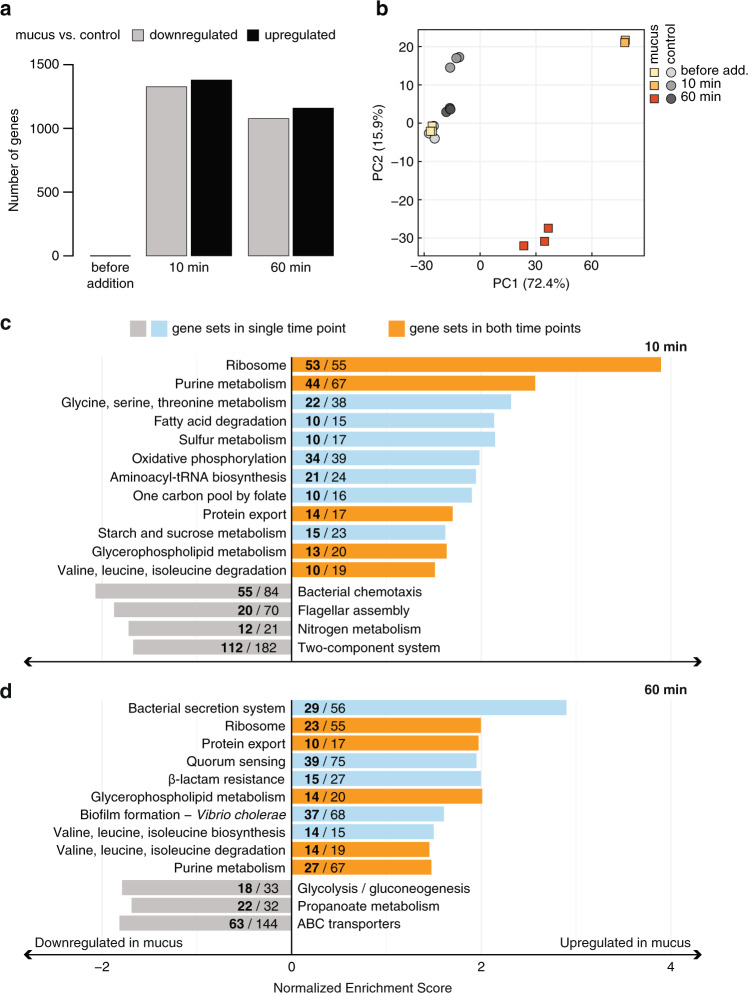
Exposure to coral mucus leads to genome-wide transcriptional shifts in *V. coralliilyticus*. **a** Number of genes that were significantly upregulated or downregulated in coral mucus at each time point (adjusted *p* < 0.01). **b** Principal component analysis (PCA) plot showing all RNA-seq samples in the 2D plane spanned by the first two principal components. PCA was performed using raw read count data after variance stabilizing transformation. **c**, **d** Gene set enrichment analyses (GSEA) on mucus vs. control at 10 min (**c**) and 60 min (**d**). Gene sets (KEGG pathways, bar labels) that were significantly upregulated or downregulated in coral mucus (FDR *q* < 0.25) are shown, and their bars are ordered top to bottom by FDR *q* values (smallest to largest; *i.e.*, most significant to least significant) within each expression category (upregulated in mucus, blue/orange, or downregulated in mucus, gray). Number of significantly differentially expressed genes (adjusted *p* < 0.01) in mucus that were included in the GSEA (bold), and the total number of genes in the *V. coralliilyticus* genome assigned to each KEGG pathway, are shown. Orange bars represent gene sets that were upregulated in mucus at both 10 and 60 min.

Sequencing and alignment of mRNA libraries to the *V. coralliilyticus* BAA-450 reference genome, which contains 5078 protein-coding sequences [34], resulted in 5020 genes with non-zero total read count (Supplementary Tables 1, 2). Coral mucus led to a genome-wide response, with significant upregulation of 1379 genes (27% of detected genes) after 10 min, and 1159 genes (23%) after 60 min, relative to controls at the same time points (Fig. 3a). In addition, coral mucus exposure was associated with significant downregulation of 1326 genes (26%) after 10 min and 1076 genes (21%) after 60 min (Fig. 3a). Of all significantly differentially expressed genes, 1521 genes (30%) were shared between 10 and 60 min (Supplementary Table 3). These results suggest that exposure to coral mucus leads to large shifts in the gene expression profile of *V. coralliilyticus*.

The changes in *V. coralliilyticus* gene expression following exposure to coral mucus occurred rapidly and on the same timescales as the chemokinetic responses. Cells exposed to mucus (10 min) displayed the largest transcriptional shift in the principal component analysis (PCA) space (first two principal components captured 88.3% of the variance; Fig. 3b). Replicate libraries possessed small sample-to-sample distances (Supplementary Fig. 8) and clustered tightly together in the PCA space (Fig. 3b), indicating little inter-replicate variance. Control and mucus cells at 10 min shared similar transcriptional shifts along the second component (PC2, 15.9%, Fig. 3b), suggesting that the transcriptional changes in control cells were also present in mucus cells. After 60 min of mucus incubation, gene expression occupied a distinct space on PC2 in comparison with other time points, indicating a potential physiological switch that requires the expression of a different group of genes compared to the early time point (10 min). Taken together, our results reveal a rapid (within 10 min) transcriptional response of the pathogen to coral mucus that mirrors the timescales of its behavioral changes.

**Fig. 4:**
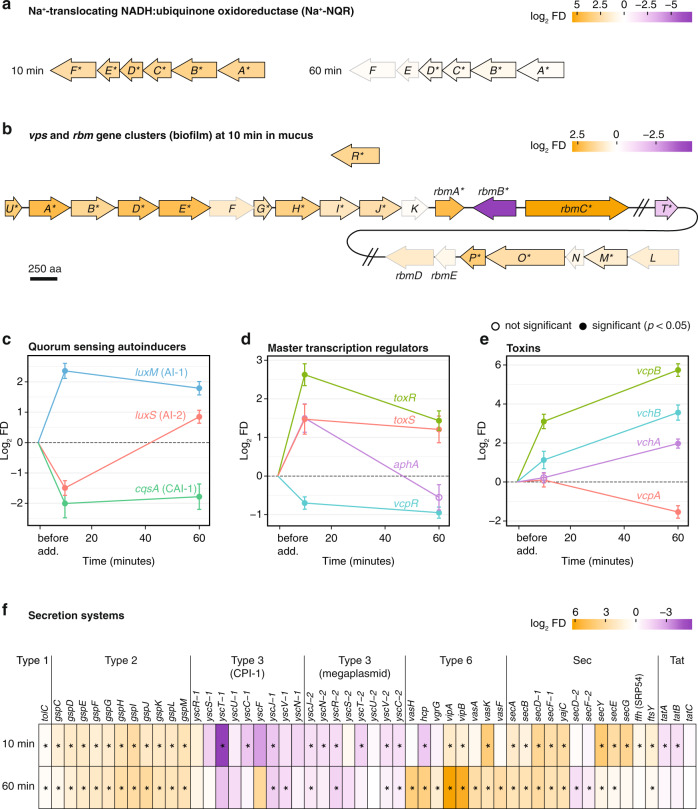
Differential expression of Na^+^-NQR enzyme, surface association, and host damage genes in coral mucus. Log_2_-transformed fold difference between mucus and control (log_2_ FD) and their adjusted *p* values were determined using DESeq2. **a** Na^+^**-**NQR genes *nqrA–F* were identified through homology with *nqr* genes of *Vibrio alginolyticus* (Supplementary Table 6). **b** *Vibrio* biofilm genes in the *vps* and *rbm* gene clusters were identified through homology with *V. cholerae* genes (Supplementary Table 7). *Nqr* genes (*nqrA–F*) (**a**) and *vps* genes (*vpsU, vpsA–P,  vpsR, vpsT*) (**b**) are labeled with their respective suffix letters. Lengths of arrows are proportional to protein size (scale bar; aa = amino acids). **c**–**e** Log_2_ FD of quorum-sensing autoinducer synthase genes (**c**), *Vibrio* master transcription regulators (**d**), and toxins (**e**) at 10 and 60 min. Homology was found with genes in other Vibrios (Supplementary Table 8). Fold difference before addition was assumed to be 1:1 (mucus:control, log_2_ FD = 0). Error bars represent standard error estimates for the log_2_ FD values. **f** Heatmap showing differential expression of secretion system genes identified through KEGG pathway assignments (Supplementary Table 10). The *V. coralliilyticus* genome has two sets of type 3 secretion genes [34]. CPI-1, Coralliilyticus Pathogenicity Island-1. Colors (**a**, **b**, **f**) indicate log_2_ FD values. Asterisks (**a**, **b**, **f**), black outlines of arrows (**a**–**b**), and filled circles (**c**–**e**) mark genes with significant differential expression (adjusted *p* < 0.05). Open circles, not significant.

Given the strong increase in swimming speed observed by video microscopy within the first 10 min of mucus exposure (Fig. 2), we searched for potential mechanisms underpinning the chemokinesis behavior in the transcriptome (Supplementary Tables 4, 5). Our results from differential expression analysis (DESeq2) revealed the upregulation of all six genes of the Na^+^-translocating NADH:ubiquinone oxidoreductase (Na^+^-NQR) enzyme in coral mucus compared to controls at the 10 min time point only (Fig. 4a, Supplementary Table 6). The Na^+^-NQR enzyme participates in the respiratory electron transport chain and is responsible for generating a sodium motive force that drives flagellar rotation in Vibrios [35, 36]. Although the rapid onset (within 2 min) of chemokinesis suggests that this behavior is not entirely a result of changes in gene expression (bacterial protein production typically takes 10 min or longer [37]), the increase in Na^+^-NQR enzyme production may have enabled the sustained chemokinesis observed over the duration of our experiments.

Surprisingly, chemotaxis genes and flagella genes were downregulated in coral mucus compared to controls at 10 min (Fig. 3c, Supplementary Fig. 9), despite the strong increase in swimming speed observed by video microscopy at the same time point (Fig. 2). Gene Set Enrichment Analysis (GSEA) revealed that chemotaxis (KEGG 02030) and flagellar assembly (KEGG 02040) gene sets were downregulated within 10 min of incubation with coral mucus (Fig. 3c, Supplementary Fig. 9). All 17 chemotaxis *che* genes (*cheA, cheB, cheD, cheR, cheV, cheW, cheY, cheZ*) were downregulated, except *cheX* (EEX30576) (Supplementary Fig. 9). The majority of the 50 methyl-accepting chemotaxis protein (MCP) genes encoded in the *V. coralliilyticus* genome were differentially expressed, with 26 downregulated and only 15 upregulated (Supplementary Fig. 9). None of the 74 flagellar assembly genes (KEGG 02040 plus four additional genes found manually) were upregulated in coral mucus at 10 min, with all 31 differentially expressed genes downregulated (adjusted *p* < 0.05; Supplementary Fig. 9). Taken together, these patterns of gene expression led us to speculate that coral mucus, while inducing a rapid and strong response of chemokinesis, may also provide a cue for the transition from a motile to a non-motile (e.g., surface-associated) lifestyle.

*V. coralliilyticus* growth was observed in coral mucus (Supplementary Fig. 10), which, similar to other animal mucus, is rich in sugars, lipids, peptides, amino acids (especially serine and threonine), and sulfur compounds [38–42]. Metabolic pathways involved in the catabolism of natural constituents of mucus were enriched in transcripts in mucus-treated cells at 10 min (Fig. 3c). Enriched pathways included metabolism of starch and sucrose (KEGG 00500), glycerophospholipid (KEGG 00564), glycine, serine and threonine (KEGG 00260), sulfur compounds (KEGG 00920), and fatty acids (KEGG 00071) (Fig. 3c, Supplementary Discussion). On the other hand, *V. coralliilyticus* transcriptomes after mucus treatment (10 min) were depleted in functions involved in the assimilation of inorganic nitrogen (nitrate and nitrite reductases in KEGG 00910; Fig. 3c), which may suggest a metabolic switch toward the utilization of mucus-derived organic nitrogen (e.g., amino acids and peptides, [39]). Almost all ribosomal protein genes (KEGG 03010) and aminoacyl-tRNA synthetases (KEGG 00970) were upregulated in coral mucus compared to controls at 10 min (Fig. 3c, Supplementary Fig. 11), which is indicative of elevated metabolism. Similarly, markers of cell growth, *ftsZ* and *rpoD* (EEX34708 and EEX34866; Supplementary Fig. 12) were upregulated in *V. coralliilyticus* cells exposed to coral mucus. This suite of changes suggests that upon exposure to the nutrient-rich coral mucus, *V. coralliilyticus* rapidly (within 10 min) and substantially alters its transcriptome to increase metabolism, protein production, and growth.

Upregulation of functions associated with quorum sensing (KEGG 02024) and biofilm formation (KEGG 05111) was observed in *V. coralliilyticus* cells exposed to coral mucus. Upregulated genes included those involved in quorum-sensing signal molecule production (*luxM*, *luxS*) and regulation (*aphA*, *vcpR*), as well as biofilm-related polysaccharide production (*vps* and *rbm* genes) (Fig. 4b–d, Supplementary Tables 7, 8). The *V. coralliilyticus* genes responsible for the production of different quorum-sensing autoinducer molecules (AI-1, AI-2, CAI-1) [34] displayed diverse but significant responses, with *luxM* (AI-1; EEX31502) upregulated and *cqsA* (CAI-1; EEX33462) downregulated in mucus at both 10 and 60 min relative to controls at the same time points (Fig. 4c). In contrast, *luxS* (AI-2; EEX35562) was first downregulated at 10 min (0.35×) and subsequently upregulated at 60 min (1.8×) in mucus relative to controls (Fig. 4c). Furthermore, the quorum-sensing master transcription regulators, *aphA* (EEX30687) and *vcpR* (EEX34823; homologous to *luxR* of *V. harveyi* and *hapR* of *V. cholerae* [43]), were up- and downregulated, respectively, which is consistent with their reciprocal behavior in which AphA represses *vcpR* expression [44, 45] (Fig. 4d). At 10 min in coral mucus, *aphA* was upregulated (2.8×) while *vcpR* was downregulated (0.5×) compared to controls (Fig. 4d). In Vibrios, quorum sensing is tightly coupled with biofilm formation [44, 46–48]. Indeed, the majority of the 18 *vps* (*Vibrio* polysaccharide) and 5 *rbm* (rugosity and biofilm structure modulator) genes, which are essential for biofilm formation in *V. cholerae* [49–52], were upregulated in coral mucus at 10 min (Fig. 4b). These early-onset expression changes of specific genes involved in quorum sensing and biofilm formation were followed by the significant upregulation of their associated pathways (39 quorum sensing genes in KEGG 02024; 37 biofilm formation genes in KEGG 05111) at 60 min (Fig. 3d). Taken together, these results suggest that upon exposure to coral mucus, *V. coralliilyticus* initiates a gene expression program for biofilm formation that may be involved in host colonization.

Virulence factors characteristic of *Vibrio* pathogens were amongst the most strongly and significantly upregulated genes in *V. coralliilyticus* incubated in coral mucus (Supplementary Figs. 13, 14). The master regulator of virulence, ToxR (EEX35320), and its associated stabilizer [53, 54], ToxS (EEX35319), were upregulated in coral mucus compared to controls at both the 10- and 60-min time points (Fig. 4d). Concurrently, several toxin genes were upregulated in coral mucus compared to controls at both time points (Fig. 4e). The important *V. coralliilyticus* virulence factor, VcpB zinc metalloprotease (EEX32371), was one of the most strongly and significantly upregulated genes in coral mucus at both 10 min (8.6×; Supplementary Fig. 13) and 60 min (53.5×; Supplementary Fig. 14). In addition, the VchA hemolysin (EEX31069) and the associated putative chaperone VchB (EEX31068), which are homologs of the primary virulence factors for *Vibrio vulnificus* [55, 56], were significantly upregulated in coral mucus at 60 min (3.9× and 11.8×, respectively) (Fig. 4e, Supplementary Fig. 14). Furthermore, the upregulation of other zinc metalloproteases (Supplementary Fig. 15) suggests the existence of multiple, as yet uncharacterized, zinc metalloproteases available to *V. coralliilyticus* (Supplementary Discussion, Supplementary Table 9). Together, these virulence factors and toxins may be responsible for the tissue lysis of corals previously observed during *V. coralliilyticus* BAA-450 infection at elevated temperatures [8].

The bacterial secretion system (KEGG 03070) and protein export (KEGG 03060) gene sets were significantly upregulated in coral mucus at 60 min (Fig. 3d), suggesting elevated secretion of proteins. In particular, types 2, 6 (T2SS, T6SS) and Sec secretion system genes were collectively upregulated in coral mucus (Fig. 4f, Supplementary Table 10). Indeed, *vipB* (EEX32048), which encodes an essential component of T6SS [57], and *sec* genes were amongst the most significantly and highly upregulated genes in coral mucus at 60 min (Supplementary Fig. 14, Supplementary Table 5). In addition, the upregulation of β-lactam resistance (KEGG 01501; Fig. 3d), as well as several of the multidrug-resistance efflux pump (*vex*) genes (Supplementary Fig. 16), may confer resistance against antibiotic compounds produced by commensal bacteria within coral mucus [58–63]. Taken together, these results suggest that exposure to coral mucus induces *V. coralliilyticus* to upregulate toxin production, secretion and antibiotic resistance genes, which may be important for host damage and for defense and competition against the commensal microbiome during host colonization.

## Discussion

We have reported a rapid behavioral and transcriptional response of *V. coralliilyticus* to coral mucus exposure, which led to a two-fold increase in swimming speed and significant differential expression of 53% of the genes in the genome within 10 min. Our findings identify coral mucus as a potential chemical signal that induces pathogens to prepare for host colonization and infection. These responses are in line with the behavioral and physiological versatility characteristic of marine copiotrophic bacteria, which are often adapted to boom and bust lifestyles [64, 65]. Yet the extent and the rapidity of the responses observed here suggest that temporally precise orchestration of behavior and gene expression is important for coral host colonization by *V. coralliilyticus*.

Chemokinesis in response to exposure to coral mucus is potentially a strategy for *V. coralliilyticus* to seize a limited window of opportunity to reach the coral surface. By increasing swimming speed, bacteria also enhance their chemotactic velocity, leading to a decrease in the time required to follow a chemical gradient to its source. This was previously shown for *V. coralliilyticus* using microfluidic gradient experiments [9, 10] and appears to be a more general feature of Vibrios, having also been observed in *V. alginolyticus* chemotaxing toward amino acids [13]. While swimming fast is expensive in the typically dilute ocean environment [66], energy is no longer limiting once nutrient-rich mucus is available. Instead, what is limiting is the window of time during which bacteria can exploit that mucus signal to reach the host. Not only can ambient water currents transport bacteria past the coral surface, but intense vortical flows produced by the corals themselves through cilia on their surfaces—moving at speeds much greater than bacterial swimming speeds—can result in rapid alternation of transport toward and away from the coral surface [25]. In this hydrodynamic environment, the colonization of a host by a bacterial pathogen is a challenging behavioral feat, where the opportunity to home in and attach to the coral surface may only last minutes or even less. The rapid behavioral response we reported here is consistent with this dynamic environment. In particular, the strong chemokinesis—where bacteria doubled their speed—is consistent with the need to reduce the time required to migrate to the coral surface once the detection of mucus indicates the presence of a coral. Furthermore, we observed that chemokinesis in response to coral mucus was almost entirely absent at a temperature at which *V. coralliilyticus* is avirulent (18.7 °C), which is consistent with the temperature dependence of chemokinesis observed in our previous study [10]. Thus, we propose that chemokinesis is a virulence trait that is important for successful host colonization by bacterial pathogens in the dynamic host surface environment.

Entry into coral mucus represents a dramatic change in nutrient exposure for *V. coralliilyticus* compared to the oligotrophic reef waters. Accordingly, *V. coralliilyticus* rapidly upregulated metabolic pathways of nutrients that are present in coral mucus, which may fuel the energetically expensive chemokinesis trait, as well as protein production (ribosome and tRNA biosynthesis) and cell growth (*ftsZ* and *rpoD*) genes that may enable rapid proliferation and confer a competitive advantage to the pathogens as they invade the coral host microbiome [67, 68]. Chemokinesis upon homogeneous addition of nutrients has been observed in other bacteria including *Rhodobacter sphaeroides* [69], *Escherichia coli* [70] and *Azospirillum brasilense* [71], and it has been speculated that this swimming speed enhancement is mediated by increasing the proton motive force that is responsible for flagellar rotation [12, 71]. In line with this, *V. coralliilyticus* exposed to coral mucus increased the expression, on a similar timescale as the chemokinesis behavior, of genes encoding the Na^+^-NQR enzyme, suggesting that regulation of periplasmic sodium levels may help control swimming speed. Thus, we hypothesize that the metabolism of mucus substrate stimulates Na^+^-NQR activity, which in turn enables sustained chemokinesis. Additional experimental work is required to test this hypothesis.

Despite the ~2× increase in swimming speed observed through video microscopy, flagellar genes were downregulated at the early RNA-seq time point (10 min). The swimming phenotype may thus persist using the existing polar flagellum, while downregulation of flagellar genes may be a strategy to prevent further replenishment of the flagellar apparatus during the transition to a non-motile phase, evidenced by the concurrent upregulation of biofilm genes. This observation has a parallel in the removal and downregulation of flagella observed in pathogens within the human mucosa, where it is speculated to be a strategy to escape immunological detection by the host, since flagella are strong inducers of pro-inflammatory signaling [72]. While corals possess innate and adaptive-like immunity [73], whether a similar dynamic occurs on the coral surface is currently not known.

Following only 10 min of exposure to coral mucus, the master regulator of *Vibrio* virulence, ToxR, and its associated protein ToxS, were upregulated. ToxR is known to be essential for coral infection by *V. coralliilyticus* [74, 75], and in other *Vibrio* pathogens the ToxR regulatory system coordinates the transcription of colonization, motility, and virulence genes in response to environmental conditions [76, 77]. These downstream effects of ToxR were indeed observed in our RNA-seq results. The temporal modulation of quorum-sensing autoinducer molecule (AI-1, AI-2, CAI-1) producers as seen in our RNA-seq data may be a strategy to coordinate metabolic and lifestyle transitions at the population level, as has been observed in *Vibrio harveyi* [78]. One such lifestyle transition may be biofilm formation, which is tightly regulated by quorum sensing in *Vibrio* pathogens [47, 79]. Indeed, we observed the upregulation of biofilm-related *vps* and *rbm* gene clusters in coral mucus at 10 min. Furthermore, we observed the upregulation of important *Vibrio* toxins, VcpB zinc metalloprotease, and VchA and VchB hemolysins, in coral mucus at both 10- and 60-min time points. Similarly, several secretion systems were upregulated, including the Sec-dependent and type 2 secretion systems, which are together responsible for extracellular secretion of a broad range of proteins, including toxins and degradative enzymes involved in the pathogenesis of many Gram-negative bacteria [80–82]. Type 6 secretion systems have been observed to be responsible for the injection of toxic effector proteins into bacterial cells in antagonistic interactions [83–85]. Taken together, the upregulation of *toxR* and *toxS*, as well as their downstream gene expression effects, suggest that coral mucus serves as an environmental signal for *V. coralliilyticus* to activate host colonization and virulence gene expression programs.

The VcpB zinc metalloprotease is a key virulence factor of *V. coralliilyticus* that causes photoinactivation of coral endosymbionts and coral tissue lesions [7], and its rank as one of the most highly and significantly upregulated genes in our RNA-seq dataset suggests that the bacterium rapidly responded to coral mucus as a cue to initiate its virulence program. However, the second zinc metalloprotease that has been implicated in *V. coralliilyticus* infections of corals, VcpA (EEX33179) [8], was downregulated in our experiment. The two zinc metalloproteases (VcpA and VcpB) may thus play redundant roles in *V. coralliilyticus* infections and may be important in different environmental contexts (Supplementary Discussion).

**Fig. 5:**
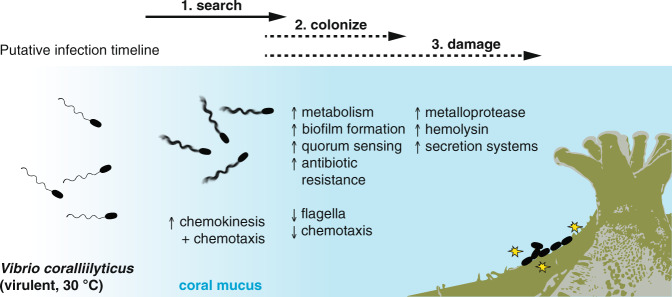
Putative infection timeline of *V. coralliilyticus*. Our results suggest that exposure to coral mucus triggers a suite of behavioral and transcriptional responses in *V. coralliilyticus* leading up to infection. Within 2 min of coral mucus exposure, *V. coralliilyticus* induces strong chemokinesis, which, coupled with chemotaxis [9, 10], allows the pathogens to reach the coral surface faster. Also early upon coral mucus exposure, upregulation of genes for metabolism of mucus components, biofilm formation, quorum sensing, and antibiotic resistance, and downregulation of flagella- and chemotaxis-related genes, enable host colonization and competition with commensal bacteria. Toxin genes (zinc metalloproteases and hemolysins; yellow stars) and secretion system genes are upregulated in coral mucus, which may lead to host tissue and symbiont damage. Solid arrows indicate bacterial responses for which we have direct observational evidence; dotted arrows indicate hypothesized phenomena based on our RNA-seq data. Figure adapted from Garren et al., 2014 [9].

Our results underscore the rapidity of behavioral and transcriptional changes that occur in a coral pathogen upon entry into the host environment (Fig. 5). These changes in swimming and gene expression patterns paint a clear sequence of events immediately preceding infection—although further validation with direct phenotypic evidence is required. Upon exposure to coral mucus, the coral pathogen *V. coralliilyticus* (known to chemotax towards coral mucus [9]) increases swimming speed by up to two-fold within minutes, a response that, in the natural environment, would lead to faster chemotaxis and a halving of the time required for the pathogen to track the coral surface from which the mucus signal originates. This capacity to rapidly chemotax into the coral surface microenvironment is important because of the short window of opportunity available to pathogens in the hydrodynamic environment surrounding corals. Simultaneously, transcriptional changes indicate that mucus exposure immediately prompts *V. coralliilyticus* to increase nutrient metabolism and prepare for host colonization and damage. The downregulation of motility genes, puzzling at first in view of the strong chemokinetic response, is in fact consistent with the upregulation of quorum sensing and biofilm formation genes, together suggesting a “final dash” to the coral surface enabled by enhanced swimming speed, followed by a rapid transition to a non-motile, coral surface-associated lifestyle. The upregulation of metabolism, growth, and antibiotic resistance genes suggests that the pathogen takes advantage of mucus as an energy source, and prepares to colonize the coral surface and compete with commensal bacteria. The upregulation of host damage genes and secretion systems responsible for toxin export suggests preparation for the infection process itself.

Precise temporal control of pathogenesis is a hallmark of *Vibrio* pathogens [86, 87], which are capable of rapidly modulating their lifestyle between free-swimming and biofilm phases in response to their environment, in particular temperature changes [34, 86, 88]. The frequency of acute temperature-rise in reef waters is increasing [89], giving additional opportunities for temperature-dependent bacterial pathogens, such as *V. coralliilyticus*, to infect corals [90, 91]. In this context, understanding the mechanisms underlying the earliest stages of bacterial infections is critical in anticipating future disease outbreaks and curbing coral mortality to protect the ecosystems that they support.

## Supplementary information

Supplementary Information

Supplementary Table 1

Supplementary Table 2

## Data Availability

The data that support the findings of this study are available from the corresponding author on request (total data size ~2 TB). Raw, filtered sequencing data reported in this paper have been deposited in the Sequence Read Archive (accession PRJNA707316).

## Code availability

All computer code (in MATLAB and R) developed for this study is available from the corresponding author on request.
