# Supplementary material for: Coral mucus rapidly induces chemokinesis and genome-wide transcriptional shifts toward early pathogenesis in a bacterial coral pathogen
Source: ISME J. 2021 Jun 24;15(12):3668–82. doi: 10.1038/s41396-021-01024-7 (PMC8630044; doi:10.1038/s41396-021-01024-7)
Supplement: Supplementary file 1 — Supplementary Information [file 41396_2021_1024_MOESM1_ESM.pdf]

Supplementary Information for

Coral mucus rapidly induces chemokinesis and genome-wide transcriptional shifts toward early pathogenesis in a bacterial coral pathogen

by

Gao *et al.*

Table of Contents

In this document, we provide supplementary information on Methods (§1), Supplementary Figures 1–16 (§2), Supplementary Tables 1–10 (§3), supplementary discussion of Results (§4), and additional references (§5).

1. Supplementary Methods 3

1.1. Analysis of microscopy videos 3

1.1.1. Cell segmentation, swimming track reconstruction, and speed calculation 3

1.1.2. Motile and non-motile cell determination 4

1.2. RNA sampling, isolation, sequencing, and sequence alignment 4

1.2.1. RNA extraction 4

1.2.2. Ribosomal RNA removal and mRNA sequencing 5

1.2.3. Sequence processing and alignment 5

1.3. Gene Set Enrichment Analysis (GSEA) 6

1.3.1. GSEA input table preparation 6

1.3.2. GSEA 6

1.4. Viscosity measurements 7

1.5. Growth curves and DAPI-stained cell counts 7

1.6. Coral mucus collection from *Acropora millepora* 8

2. Supplementary Figures 9

3. Supplementary Tables 28

4. Supplementary Discussion 40

4.1. Investigation of swimming speed enhancement by control cells 40

4.2. Gene expression patterns that point to virulence 40

4.2.1. Release from phosphate starvation 40

4.2.2. Evidence for *V. coralliilyticus* growth and physiological transformation in coral mucus 41

4.2.3. *vps* and *rbm* (biofilm) gene expression 41

4.2.4. Zinc metalloproteases 42

5. Additional References 44

1. Supplementary Methods

1.1. Analysis of microscopy videos

1.1.1. Cell segmentation, swimming track reconstruction, and speed calculation

Each frame of a video was processed independently for bacterial cell segmentation. First, images were smoothed by applying a Gaussian spatial filter to reduce the background signal noise and increase contrast. Next, putative cell-containing pixels were recognized by applying a threshold on pixel intensity (determined manually for each experiment) to phase contrast images, in which cells appear as dark (i.e., low intensity) pixels. Images were converted to binary by assigning 0 to putative cell-containing pixels and 1 to all other pixels. Putative cell-containing pixels that were in contact with each other were grouped together as putative cells (particles). To remove unwanted particles, thresholds on diameter and average pixel intensity of particles were applied. Threshold values were manually determined through visual inspection for each experiment, and typically categorized particles as cells if they possessed 1 μm ≤ diameter ≤ 4 μm, and particle intensity ≤ –500 a.u.

Individual trajectories (*x,y*-coordinates vs. time), each representing the swimming path of a single cell, were reconstructed from identified cell positions through subsequent frames using a particle tracking routine. Cells tracked for less than four consecutive frames (< 0.1 s) were excluded from trajectory reconstruction, as were cells with displacements exceeding 6 μm between two consecutive frames (equivalent swimming speed of ≥ 180 μm/s). The success of cell segmentation and swimming track reconstruction was manually assessed by visualizing 40 frames of each video with swimming track information overlaid.

A swimming trajectory was sometimes inappropriately broken into two or more tracks, for example due to a motile cell going into and out of focus between frames. To reconnect broken trajectories, the future position of a bacterium was predicted using its direction and speed (i.e., velocity) at the end of its track, which was locally smoothed over 3 frames (0.1 s) using a linear fit. Subsequently, if another track with a similar velocity began within 8 μm distance from the end of the first track, and within the next 1–3 frames, the two tracks were reconnected as one. Application of this trajectory reconnection algorithm led to an average reduction to 83% ± 6% (mean ± s.d.) of the original number of tracks.

Finally, we discarded short swimming tracks from further analyses by applying a minimum track length threshold of 10 frames (0.33 s), inclusive, to enable accurate calculation of swimming speeds. From these reconstructed trajectories that passed the minimum track length threshold, we calculated the swimming speed of each cell by averaging the instantaneous speed over the duration of that cell's trajectory. The mean speed of the population was quantified by averaging over all trajectories detected in each microscopy video, representing a single time point in mucus or control condition.

1.1.2. Motile and non-motile cell determination

To differentiate between motile and non-motile cells, two types of thresholds (speed and extent of ballistic motion) were applied to each swimming trajectory. First, slow bacteria (median instantaneous speed < 10 μm/s) were categorized as non-motile cells. The median instantaneous speed, rather than the mean, was used to avoid disproportionate influence of extreme instantaneous swimming speeds, which are more likely to occur in longer swimming tracks. Subsequently, we fine-tuned the categorization of motile and non-motile cells by characterizing their motility as ballistic or diffusive.

Motile bacteria display ballistic motion over short time intervals, while non-motile bacteria display diffusive motion. To determine the type of motility (ballistic or diffusive) of a bacterium, we calculated the mean squared displacement (MSD) as a function of short time intervals (Δt) ranging from 1 frame (0.03 s) to 9 frames (0.3 s) at 1-frame intervals, and quantified the exponent α of this dependence (MSD ~ Δt^{α}). The maximum Δt of 9 frames (0.3 s) was chosen as a time window that is short enough to only observe one ballistic motion, but long enough to differentiate between diffusive and ballistic motion. The MSD of particles such as bacterial cells is described by a power law, (MSD ~ Δt^{α}), where $\alpha = 2$ for ballistic motion, $\alpha = 1$ for diffusive motion, and $\alpha = 0$ for non-motile cells. Thus, we determined the MSD exponent, α, for each bacterium as the slope of the linear fit of $\log_{10}$-transformed Δt and MSD.

Since *V. coralliilyticus* bacteria do not swim ballistically throughout their entire trajectories, but rather display a run-reverse-flick mode of swimming with intermittent ballistic motion (1,2), the MSD exponent of motile cells is expected to fall in the range $1 < \alpha \leq 2$. Thresholds were determined by inspection of the scatter plot of median instantaneous velocities and MSD exponents of all swimming tracks (Supplementary Fig. 1). Subsequently, cells with MSD exponent $\alpha \geq 1$, in addition to median instantaneous speed ≥ 10 μm/s, were categorized as motile. A sensitivity analysis was performed that showed how the precise value of the selected parameter thresholds did not substantially affect results (Supplementary Fig. 2).

1.2. RNA sampling, isolation, sequencing, and sequence alignment

1.2.1. RNA extraction

For total RNA extraction, each sample was thawed, the closed tip of the filter cartridge was cut open, and the RNAlater solution was removed by filtration through the membrane by attaching a syringe. The filter cartridge was then broken in a sterile Whirl-Pak plastic bag using a hammer to access the filter membrane containing RNA-stabilized cells. The filter membrane was cut into pieces and placed in Eppendorf tubes containing 15 mg/ml lysozyme, vortexed, and incubated at 37 °C for 10 min. Samples were bead-beaten with sterile autoclaved beads for 10 min, with brief submersion of the sample-containing tubes in ice at 2-min intervals to keep cool. Tubes were centrifuged at 1,000 rpm in a microcentrifuge for 1 min and the supernatant was collected into new tubes, which were centrifuged at 14,000 rpm for 3 min. To each pellet, 1 ml TRIzol was added, and the tube was vortexed for 1 min for pellet dissolution, then incubated at room temperature for 5 min. Next, 200 μl chloroform was added to partition RNA into the aqueous supernatant for separation, vortexed to mix, then incubated

for 8 min. After centrifuging at 12,000×*g* for 15 min, the aqueous phase was removed. To increase precipitation efficiency from the dilute RNA solutions, 1 µl glycogen (15 mg/ml) was added, followed by 0.5 ml isopropanol to precipitate total RNA. After overnight incubation at −80 °C, samples were centrifuged at 14,000 rpm for 30 min. Pellets were washed with 70% ethanol, centrifuged at 12,000×*g* for 5 min, and air-dried. Extracted total RNA pellets were resuspended in 50 µl DEPC-treated RNase-free water and stored at −80 °C. The coral mucus-only RNA extraction control did not yield any detectable RNA.

1.2.2. Ribosomal RNA removal and mRNA sequencing

Ribosomal RNA was removed from total RNA using Ribo-Zero rRNA Removal Kit for bacteria (Epicentre Biotechnologies). Due to low RNA yield, all samples from replicates 1 and 2 were sequenced using the ultra-low input protocol (> 10 ng total RNA), while replicate 3 samples were sequenced using the low input protocol (> 100 ng total RNA). The sample-to-sample distance heatmap showed clustering of samples according to time point and condition, confirming that the different sequencing protocols (ultra-low or low inputs), did not affect our RNA-seq results (Supplementary Fig. 8). Due to RNA degradation, the mucus-treatment sample at 10 min from replicate 2 could not be sequenced. Thus, 17 samples in total were sequenced.

Stranded cDNA libraries were generated using the Truseq Stranded RNA LT kit (Illumina). The rRNA-depleted RNA was fragmented and reverse transcribed using random hexamers and SSII (Invitrogen) followed by second strand synthesis. The fragmented cDNA was treated with end-pair, A-tailing, adapter ligation, and 10 cycles (15 cycles for the ultra-low input protocol) of PCR. The prepared libraries were quantified using KAPA Biosystem's next-generation sequencing library qPCR kit and run on a Roche LightCycler 480 real-time PCR instrument. The quantified libraries were then prepared for sequencing on the Illumina HiSeq sequencing platform using a TruSeq paired-end cluster kit, v4, and Illumina's cBot instrument to generate a clustered flow cell for sequencing. Sequencing of the flow cell was performed on the Illumina HiSeq2500 sequencer using HiSeq TruSeq SBS sequencing kits, v4, following a 2x100 indexed run recipe.

1.2.3. Sequence processing and alignment

Raw FASTQ file reads were filtered and trimmed using the JGI QC pipeline. Using BBDuk (www.sourceforge.net/projects/bbmap), raw reads were evaluated for artifact sequences by kmer matching (kmer = 25), allowing 1 mismatch. Detected artifacts were trimmed from the 3' ends of the reads. RNA spike-in reads, PhiX reads, and reads containing any Ns were removed. Quality trimming was performed using the Phred trimming method set at Q10. Following trimming, reads under the minimum length threshold of 45 bases were removed.

Raw reads from each library were aligned to the reference genome (*V. coralliilyticus* ATCC BAA-450, NCBI Taxon ID 675814) using BWA (3) with only unique mapping allowed (BAMs/directory). If a read mapped to more than one location, it was excluded. As a result, 99.48% (5022 genes) of the filtered FASTQ reads mapped to the *V. coralliilyticus* reference genome. featureCounts (4) was used to generate the raw gene counts. Counts refer to fragments: for example if the library was a paired-end

run where both reads aligned to the same feature/gene in the reference genome then it would be represented as a count of 1 in the raw gene counts.

1.3. Gene Set Enrichment Analysis (GSEA)

1.3.1. GSEA input table preparation

The KEGG Pathway assignment database for genes (as KEGG Orthology (KO) identifiers) of *V. coralliilyticus* OCN014 (code vct) was obtained from Kyoto Encyclopedia of Genes and Genomes (www.genome.jp/kegg). No KEGG Pathway assignment database for *V. coralliilyticus* BAA-450 was available. According to this KEGG Pathway assignment database, there are 116 metabolic pathways with at least one gene assignment from the *V. coralliilyticus* genome.

The database containing *V. coralliilyticus* ATCC BAA-450 reference genome information, including KO number assignments of genes, was obtained from the JGI IMG website (https://img.jgi.doe.gov). According to the JGI dataset, 2355 genes in the *V. coralliilyticus* BAA-450 genome did not have a KO number assigned. Most of these genes with no KO number assignment were annotated as "hypothetical proteins" (1089 genes). Of the remaining 2789 genes with KO number assignments, 1375 were not assigned to any KEGG pathways. This may be due to the difference in the strains of *V. coralliilyticus* between the KEGG (OCN014) and JGI (BAA-450) databases, but is more likely due to inadequate information available on the genes for pathway assignment. As a result, 1414 genes that were assigned to at least one KEGG Pathway were included in the GSEA gene matrix table (.gmt file). The majority of the genes (888 genes) were assigned to only one KEGG Pathway. The KEGG Pathways with the greatest number of gene assignments were "two-component system" (KEGG 02020, 182 genes); "ABC transporters" (KEGG 02010; 144 genes); "bacterial chemotaxis" (KEGG 02030; 84 genes); "quorum sensing" (KEGG 02024; 75 genes); and "flagellar assembly" (KEGG 02040; 70 genes).

The ranked gene list (.rnk file) for GSEA was built for each pairwise comparison (e.g., mucus vs. control at 10 min) using the DESeq2 analysis results (containing $\log_2$ fold difference and adjusted p values). The $\log_2$ fold difference values, which are biologically meaningful, were used to rank the genes. To give validity to the $\log_2$ fold difference values in the ranked gene list, only genes with significant differential expression (adjusted $p < 0.01$) were included in the ranked gene list. Thus, the size of genes sets (i.e., number of genes in the gene set) varied amongst different pairwise gene set enrichment analyses.

1.3.2. GSEA

The two tables (.gmt and .rnk files) were loaded into the GSEA software (v4.0.3) (5,6), which automatically excluded genes that were not present in the ranked gene list from gene sets. After this exclusion criterion is applied, gene sets that contained less than 10 genes were excluded from the analyses to enable accurate calculation of Enrichment Scores (ES). The following settings were used in the GSEA software: number of permutations (for statistical significance assessment of enrichment score) = 1000; scoring scheme $p = 1$ (weighted); normalization mode = meandiv (default method for

normalization of Enrichment Scores across analyzed gene sets, resulting in Normalized Enrichment Score, NES). NES is the primary statistic for examining gene set enrichment results. Given the large variability expression datasets and the small number of gene sets being analyzed, a relatively inclusive FDR *q*-value cutoff of 0.25 was used to assess significance. All KEGG Pathways that were significantly enriched (FDR $q < 0.25$) are presented in Figure 3.

1.4. Viscosity measurements

Viscosity measurements were performed at 25.1 °C using a VISCOlab3000 (Cambridge Viscosity) viscometer with a 0.5–10 cP range piston. Estimated error determined by the instrument was 0.3% for mucus, and 2.9% for filtered artificial seawater (FASW).

1.5. Growth curves and DAPI-stained cell counts

Growth curves of *V. coralliilyticus* in coral mucus or 1% marine broth (Supplementary Fig. 10) were measured in a sterile clear flat-bottom 48-well plate (Falcon), containing 200 μl volume per well. Coral mucus was collected on two separate days from the same *P. damicornis* coral colony and preserved at –80 °C until experimentation. Mucus was pooled and filter-sterilized (0.2 μm) before inoculation. Each growth condition was prepared in triplicates, with corresponding blank wells in triplicates (1% marine broth) or duplicates (mucus). Several bacterial colonies were picked from a marine broth agar plate, washed twice and suspended in 200 μl FASW for starvation at 30 °C for 6.5 h before it was used for inoculation. Each well was inoculated with 1 μl of prepared bacteria. The 48-well plate was incubated at 30 °C, and optical density was measured at 720 nm (OD_{720}) every 1 h (30 sec of slow orbital shaking before each time point) for 41 h using a Synergy HTX Multi-Mode Microplate Reader (BioTek Instruments).

DAPI staining followed by cell counts were performed to determine concentrations of cells associated with the OD_{720} values. Bacteria were diluted with FASW with a known dilution factor and preserved in 2% final concentration (v/v) of formaldehyde (0.2-μm-filtered). Preserved cells were kept at 4 °C until staining. Preserved cells were filtered onto a 0.2μm, 25 mm polycarbonate filter (Millipore GTBP02500) whose black color is ideal for fluorescence imaging. The DAPI dye is mixed with mounting medium (VECTASHIELD) to a final concentration of 2 μg/ml. The filter containing preserved cells was carefully touched with the DAPI-mounting medium mixture and secured between a glass slide and cover slip. Images were taken using epifluorescence microscopy with 395 nm LED excitation (10% power; Lumencor), DAPI filter cube (350/50 nm excitation; 400 nm longpass dichroic mirror; 420 nm longpass emission), 60× oil objective (CFI Plan Apochromat, numerical aperture 1.40, Nikon), and an sCMOS camera (30 ms exposure; Andor Zyla 4.2; 6.5 μm pixel). Fluorescence images were taken at ten different locations on the filter. Cells were counted manually within a cropped (400 pixels × 400 pixels) region of each image, and the mean ± s.d. cell concentration of images was calculated.

1.6. Coral mucus collection from *Acropora millepora*

Coral mucus of *Pocillopora damicornis* was used in all experiments except in a single experiment, presented in Supplementary Fig. 4, in which the effect of temperature on chemokinesis was tested. In this experiment (Supplementary Fig. 4), mucus of *Acropora millepora*, a coral species that is also vulnerable to *V. coralliilyticus* infection (7) and to whose mucus *V. coralliilyticus* performs chemotaxis (8), was used, following the same mucus collection and preservation methods as described for *P. damicornis* in the main text (mucus from five colonies was collected over three consecutive days and snap frozen each day in separate tubes).

2. Supplementary Figures

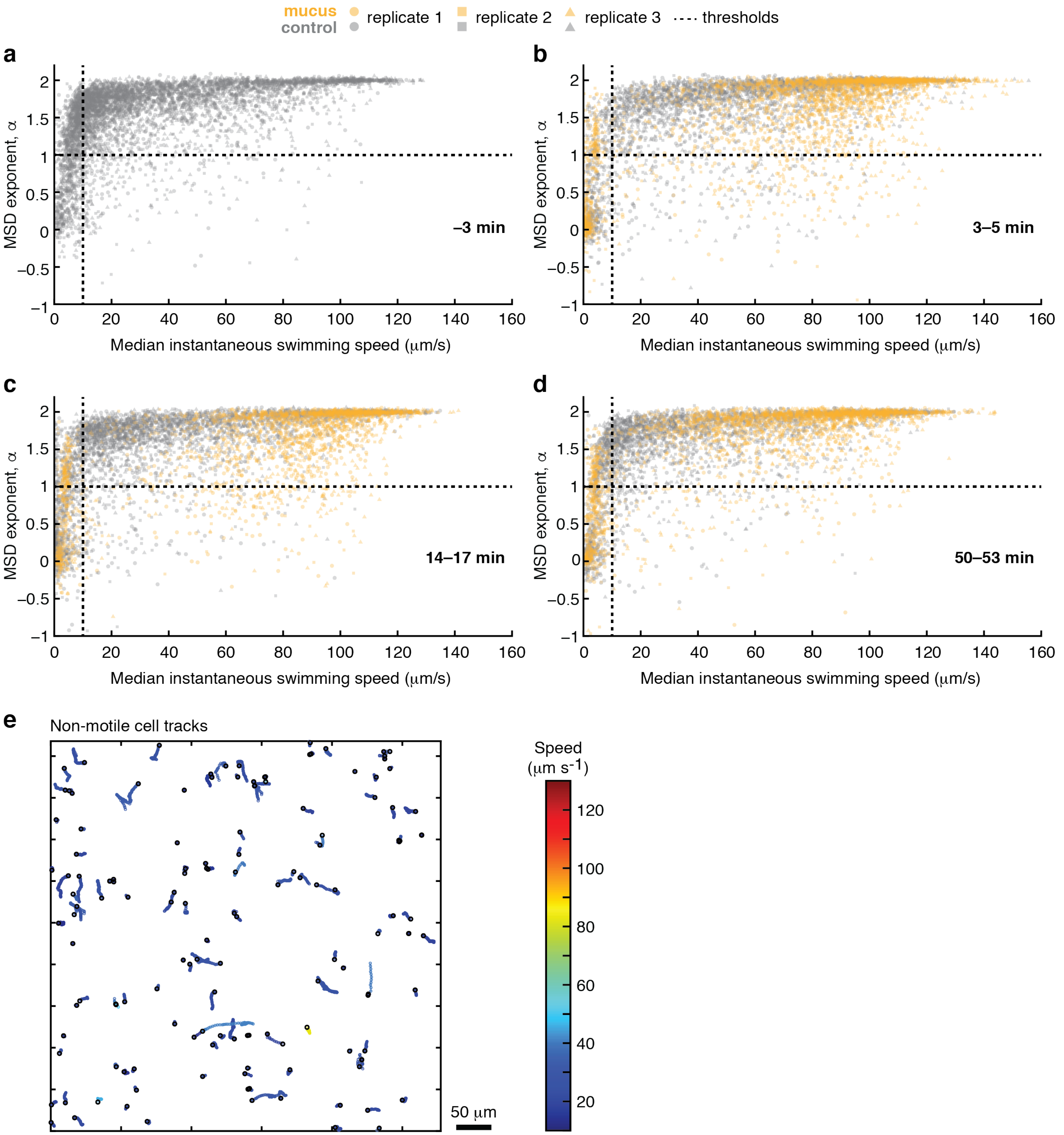

Supplementary Figure 1 | Threshold value selection for motile and non-motile cell determination. (**a–d**) Each point represents a single cell's trajectory (before motile or non-motile differentiation), whose median instantaneous swimming speed (*V*) and MSD exponent (α) are plotted. Panels represent different time points during the experiment, relative to addition of mucus (orange) or filtered spent medium (control, gray) at t = 0 min. Before addition (t = −3 min), cells designated for mucus-exposure are not differentiated from control cells (**a**). Dotted lines indicate final threshold values used for motile and non-motile cell determination; median V = 10 μm/s; α = 1. (**e**) Trajectories of non-motile cells determined by the application of final thresholds. A sample of 150 non-motile tracks that are 15 frames or longer (≥ 0.5 s) are presented (from replicate 1, at t = −4 min). Colors indicate track swimming speed on a scale that is equal to the color scale shown in Fig. 2a–b.

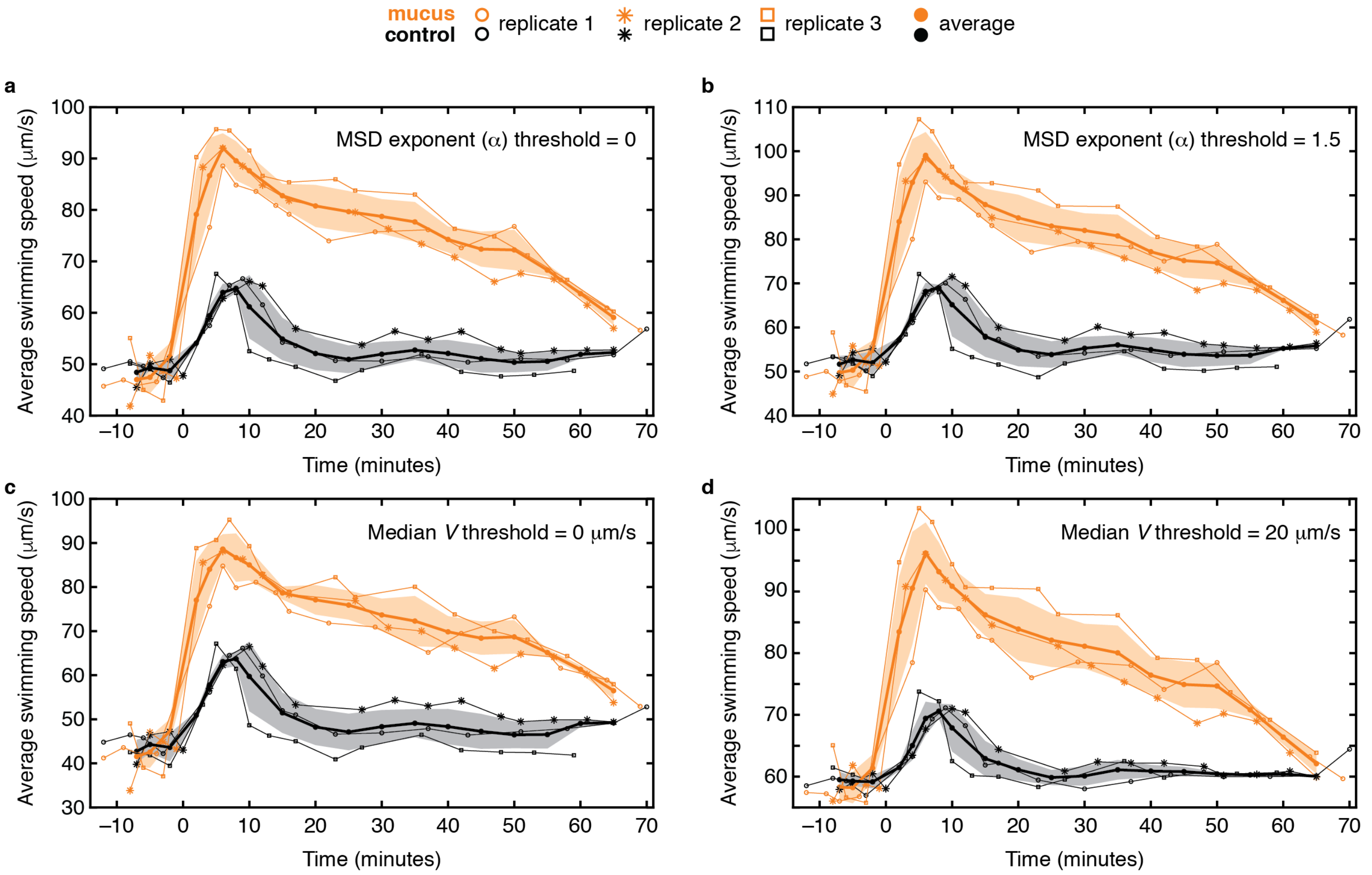

Supplementary Figure 2 | Effect of threshold value on swimming speed calculation. In each panel, the video microscopy data presented in Figure 2 were analyzed as described in Methods and Supplementary Methods, but while varying a single threshold value (α or median V) used to classify bacteria as motile or non-motile. The minimum threshold on MSD exponent α ($\alpha \geq 1$ in Figure 2) was changed to 0 in (**a**) and 1.5 in (**b**). The minimum threshold on median instantaneous swimming speed (V) (median $V \geq 10$ μm/s in Figure 2) was changed to 0 μm/s in (**c**) and 20 μm/s in (**d**). While the range of average swimming speeds (y-axis) changed slightly, the main features of the speed time series were conserved regardless of the threshold values applied.

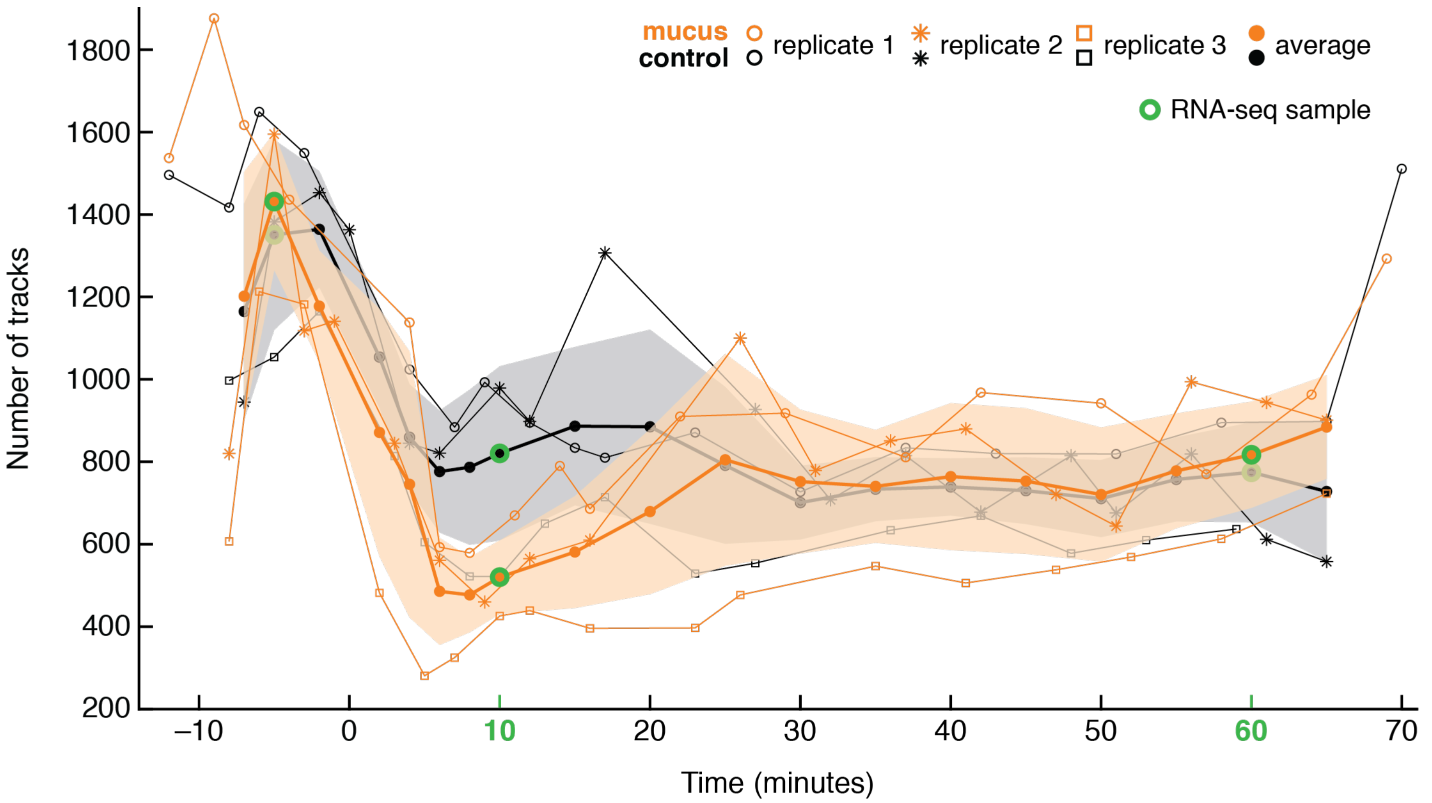

Supplementary Figure 3 | Number of motile cell tracks. Number of swimming tracks of motile cells that were included in the video analyses and whose results are presented in Fig. 2. Shaded regions represent s.d. of replicates.

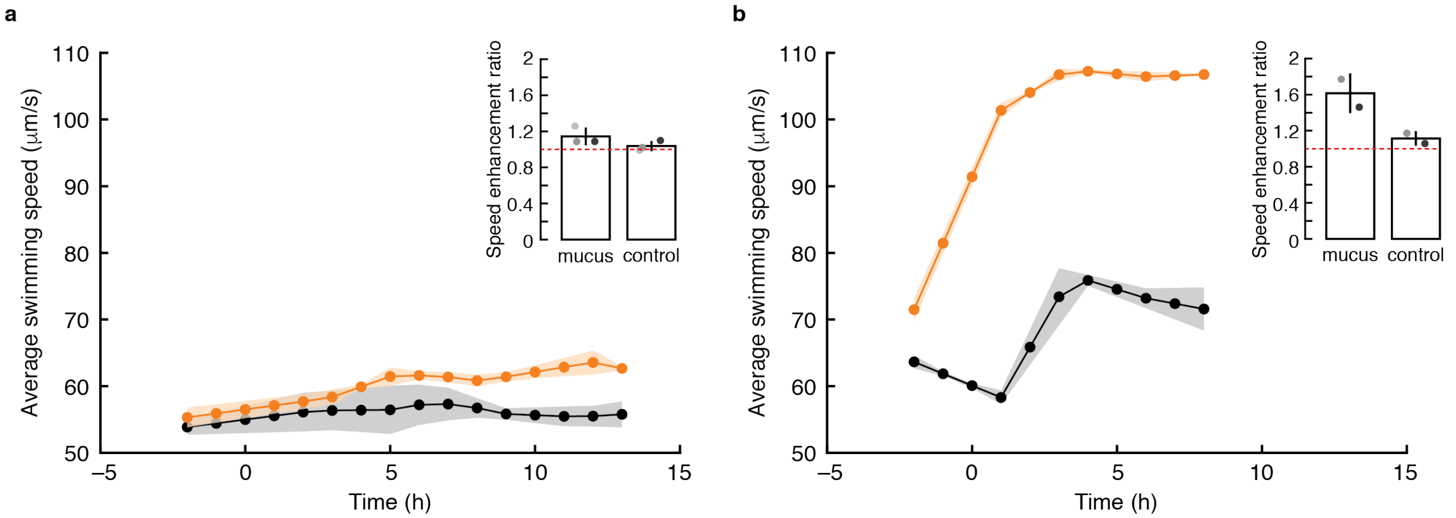

Supplementary Figure 4 | Chemokinesis is attenuated at 18.7 °C compared to 30 °C. *V. coralliilyticus* cultures were grown as described in Methods. At *t* = 0 min, filtered spent media (gray, control) or *Acropora millepora* coral mucus (orange) were added to cell cultures at 1:1 ratio (v/v) and incubated and imaged at 18.7 °C (room temperature, **a**) or 30 °C (**b**). Insets show the speed enhancement ratios between the swimming speeds at a post-addition (*t* = 5–8 min) time point vs. a pre-addition (*t* = –2 min) time point (red dotted line marks ratio of 1). Shaded regions (and error bars in inset) represent s.d. of replicates, of which there were three (**a**) or two (**b**) (data points, inset). This represents the only experiment in which *A. millepora* coral mucus was used in this study.

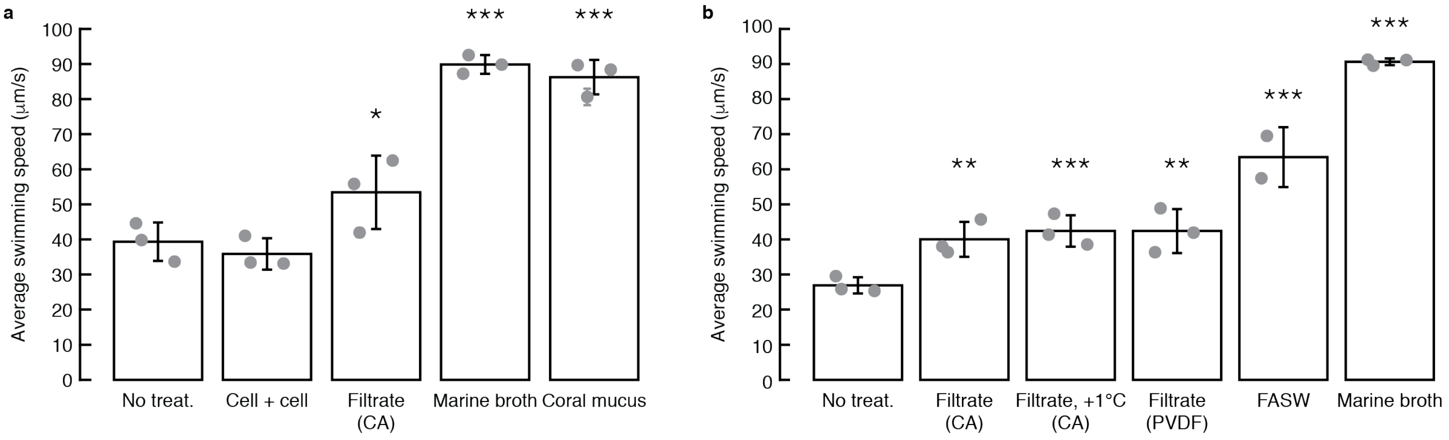

Supplementary Figure 5 | Chemokinesis in filtered spent media, sea water, and marine broth. Panels (**a**) and (**b**) represent two separate experiments, both conducted at 30 °C. All overnight culture conditions (1% marine broth), filtered spent media preparation (~20 ml through 0.2 µm filters), and imaging setups were the same as the experiment described in the main text (Methods). Treatments were performed by adding 50 µl of each specified solution to 50 µl of cells, followed by repeated pipetting to mix. Treated cells were transferred to a microfluidic device for microscopy observation 5 min after treatment. For the “Cell + cell” condition, bacteria were aliquoted into two tubes and subsequently combined again in order to test whether the act of pipetting would cause a speed enhancement. Filtered spent media (“filtrate”) were produced using two different 0.2 µm membrane materials (cellulose acetate (CA) or polyvinylidene fluoride (PVDF)) and filtration surface areas (2.8 cm^2 (CA) or 10 cm^2 (PVDF)). Higher membrane surface areas are expected to reduce the filtration pressure and thus decrease the frequency of cell-bursting that would release intracellular materials into the filtrate. Filtered spent media enhanced swimming speeds consistently (**a**,**b**), and to the same extent regardless of membrane type (CA or PVDF) or a small temperature increase (+1 °C) (**b**; two-tailed *t*-tests amongst filtrate conditions, $p > 0.01$). Chemokinesis was induced to the same extent by both marine broth and coral mucus (**a**; two-tailed *t*-tests, $p > 0.01$). Data points represent replicate treatments performed on different aliquots of cells (error bars = s.e.m. of cells; error bars may be smaller than data points). Bars and error bars represent mean and s.d. of replicate treatments. Two-tailed *t*-tests were performed on all treatment conditions compared to the no treatment condition, with statistical significance indicated by asterisks: * $p < 0.1$; ** $p < 0.05$; *** $p < 0.01$. FASW = filtered (0.2 µm) artificial sea water.

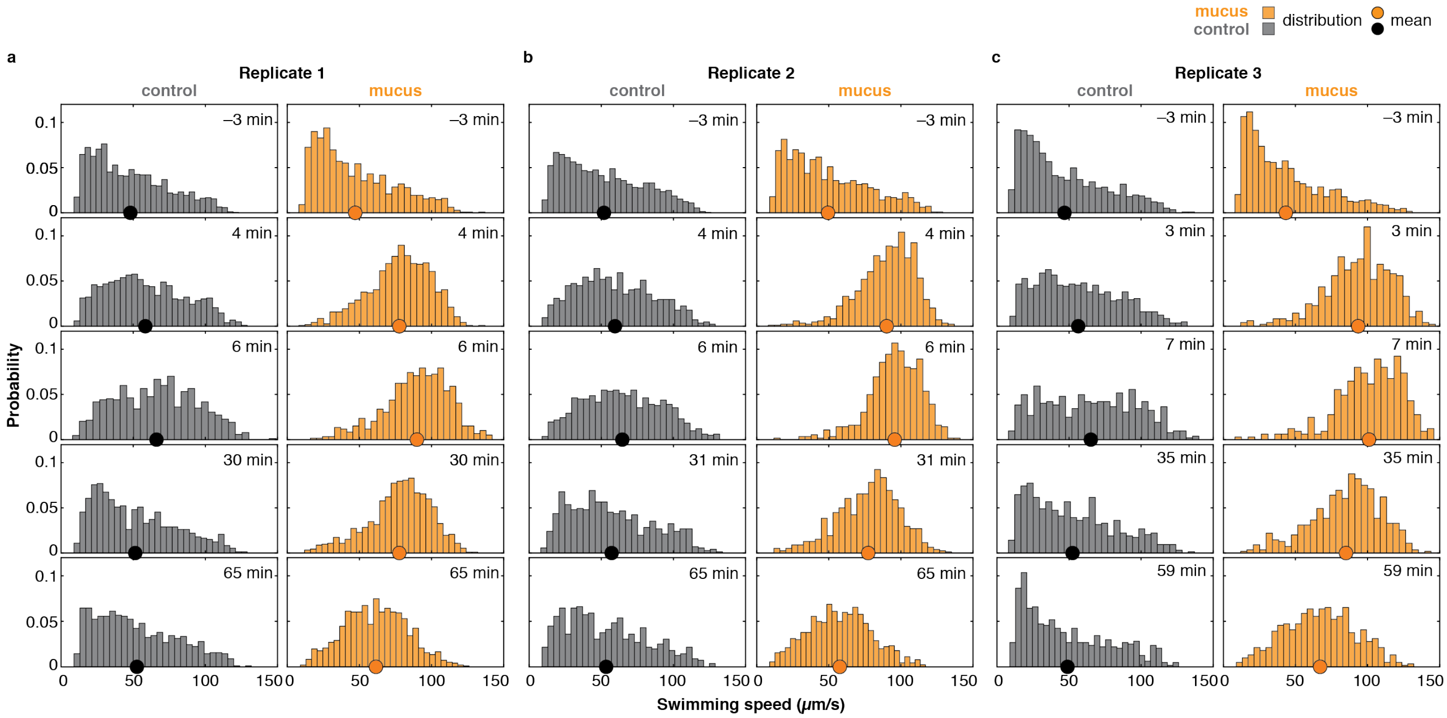

Supplementary Figure 6 | Probability distributions of swimming speeds of motile cells over time. Only motile cells were included in the analysis. Probability distributions of swimming speeds at different time points in replicate experiments 1 (**a**), 2 (**b**), and 3 (**c**) (number of bins = 30). Solid circles represent the average swimming speed of each population. Time points are relative to the addition of mucus or filtrate at *t* = 0 min.

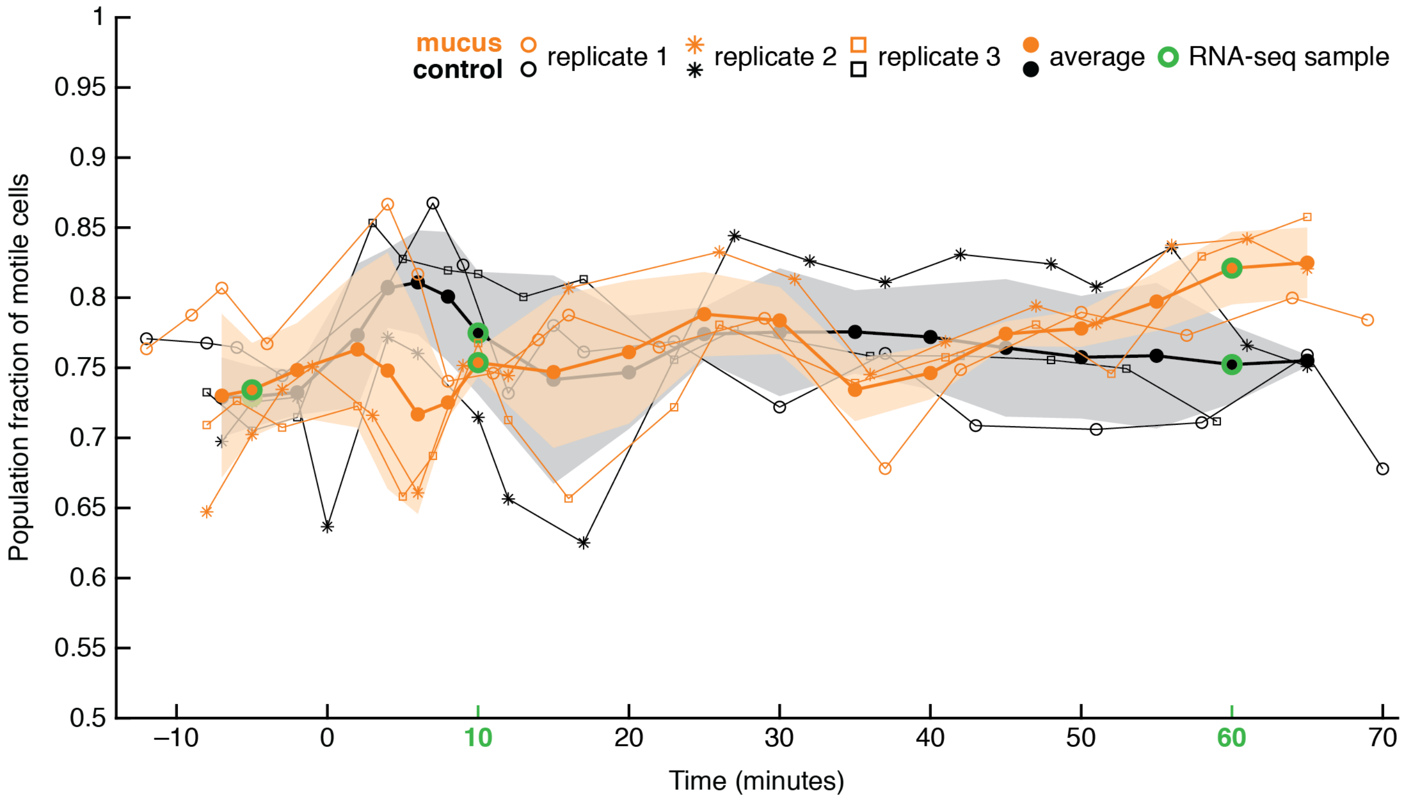

Supplementary Figure 7 | Population fraction of motile cells over time. The motile population fraction was calculated by dividing the number of motile cells by the total number of cell trajectories detected in each microscopy video. At all post-addition time points, neither mucus nor control populations deviated significantly from the pre-addition (control at $t = -2$ min) motile fraction value (two-tailed t-tests, $p > 0.01$). Furthermore, mucus and control populations did not differ significantly from each other at any time point (two-tailed t-tests, $p > 0.01$). Shaded regions represent s.d. of replicates.

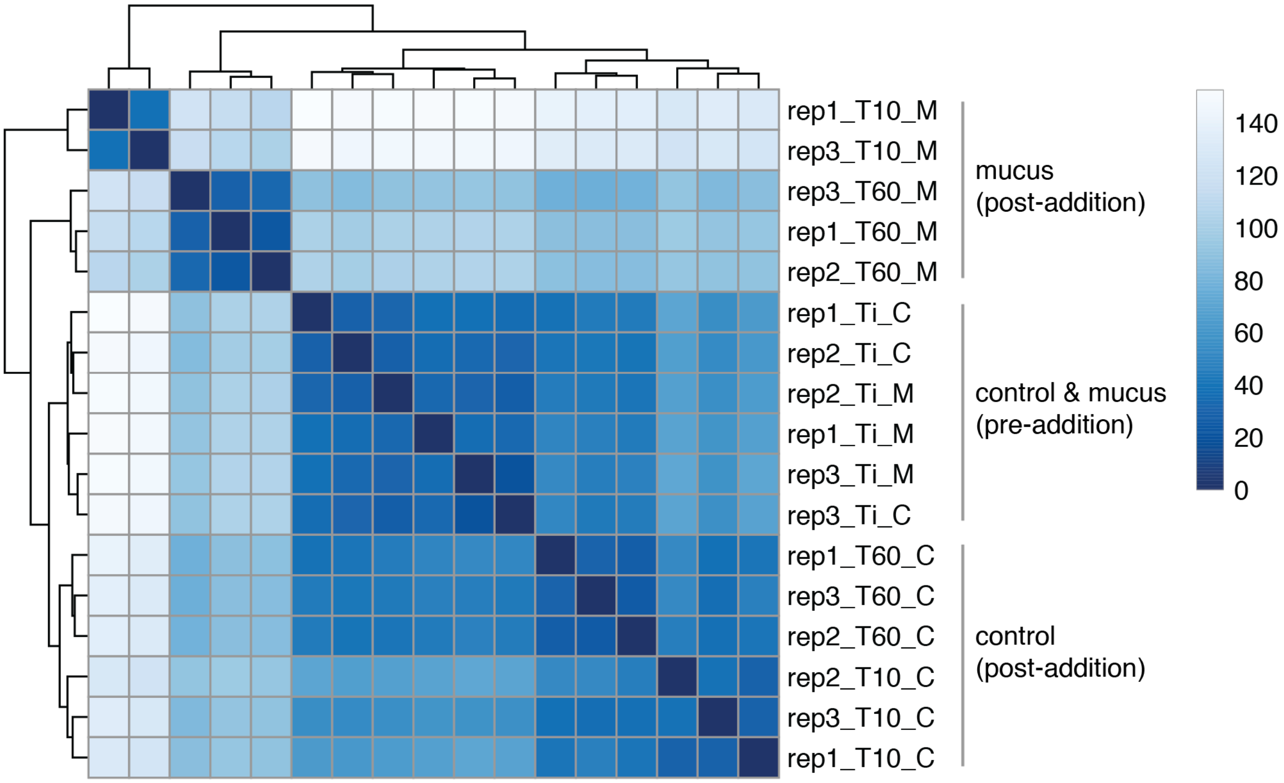

Supplementary Figure 8 | Heatmap of sample-to-sample distances. Hierarchical clustering based on sample distances calculated using raw transcript count data that were transformed with the variance stabilizing transformation (VST) method (Methods). The color scale represents sample-to-sample distances. Each row is labeled with the sample name with the following convention: rep, replicate (1–3); T, time point (i, before addition; 10, 10 min; 60, 60 min); M, mucus; C, control.

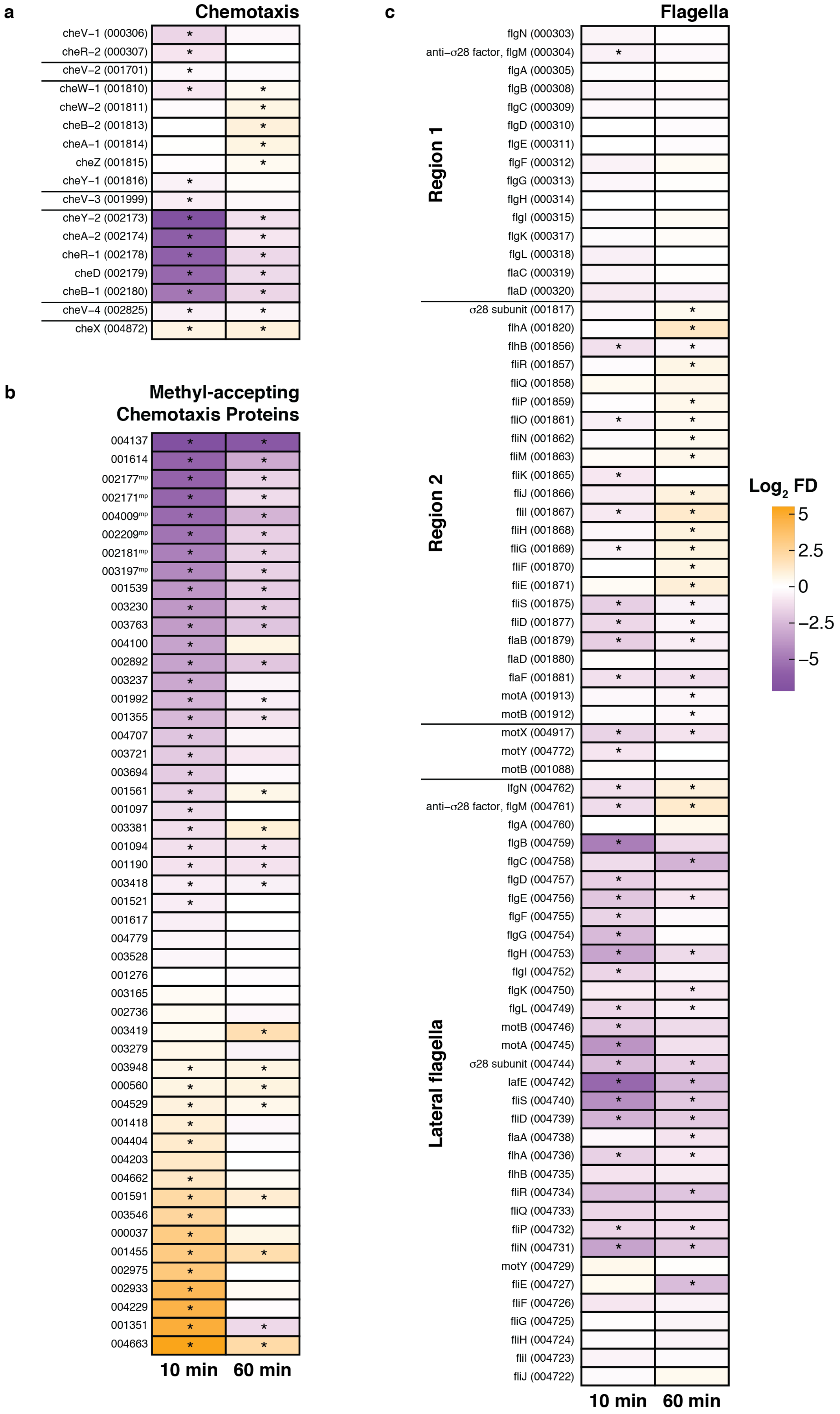
a
Chemotaxis
cheV−1 (000306)
cheR−2 (000307)
cheV−2 (001701)
cheW−1 (001810)
cheW−2 (001811)
cheB−2 (001813)
cheA−1 (001814)
cheZ (001815)
cheY−1 (001816)
cheV−3 (001999)
cheY−2 (002173)
cheA−2 (002174)
cheR−1 (002178)
cheD (002179)
cheB−1 (002180)
cheV−4 (002825)
cheX (004872)
b
Methyl-accepting Chemotaxis Proteins
c
Flagella
Region 1
Region 2
Lateral flagella
Log2 FD
5
2.5
0
−2.5
−5
10 min
60 min

Supplementary Figure 9 | Heatmaps of motility- and chemotaxis-related gene expression. Log_2-transformed fold differences between mucus and control (log_2 FD) and their adjusted *p* values were determined by using DESeq2. Columns represent time points, colors indicate log_2 FD, and asterisks mark genes with significant differential expression (adjusted $p < 0.05$). Numbers in row labels represent NCBI locus tags, and follow the prefix, "VIC_". Horizontal dividers mark putative operons. Chemotaxis *che* genes (**a**, 17 genes) and methyl-accepting chemotaxis protein (MCP) genes (**b**, 50 genes) are subsets of the bacterial chemotaxis KEGG 02030 pathway gene assignment. The methyl-accepting chemotaxis protein (MCP) genes are ranked by log_2 FD values at 10 min. Superscripts "MP" in row labels mark the six MCP genes located in the *V. coralliilyticus* megaplasmid. Flagella genes (**c**) were identified through flagellar assembly KEGG 02040 pathway gene assignment (70 genes) in addition to four manually identified genes (VIC_004917, VIC_004772, VIC_004729, VIC_004722). Putative flagellar regions 1 and 2 were determined by comparison with the *V. parahaemolyticus* genome (9). Putative lateral flagella region (10), VIC_004722–VIC_004762, is also marked.

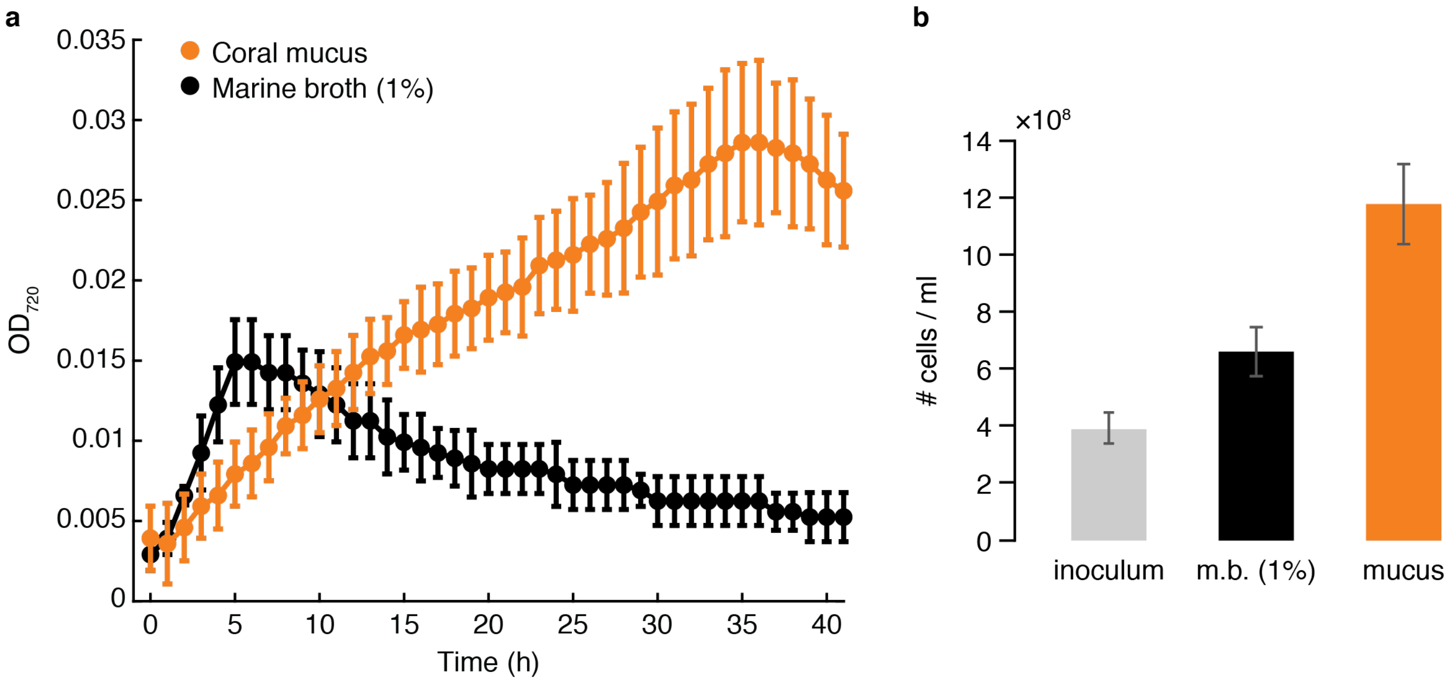

Supplementary Figure 10 | Growth curves of *V. coralliilyticus* in coral mucus and marine broth (1%) at 30 °C. (**a**) Optical densities (OD_{720}) of bacteria-free blank wells were subtracted before averaging over triplicate wells. Error bars are s.d. of triplicate wells. (**b**) Since OD measurements may be affected by bacterial attachment to walls, cells were sampled from the growth curve setup at the first (inoculum) and last time points for cell counts. DAPI-stained cells were counted, and cell concentrations were determined at inoculum (gray), and at the last time point of the growth curves for 1% marine broth (m.b., black) and coral mucus (orange). Bars and error bars are mean and s.d. of microscopy images (n = 10 images for inoculum and m.b.; n = 20 images for mucus). Detailed methods are in Supplementary Methods.

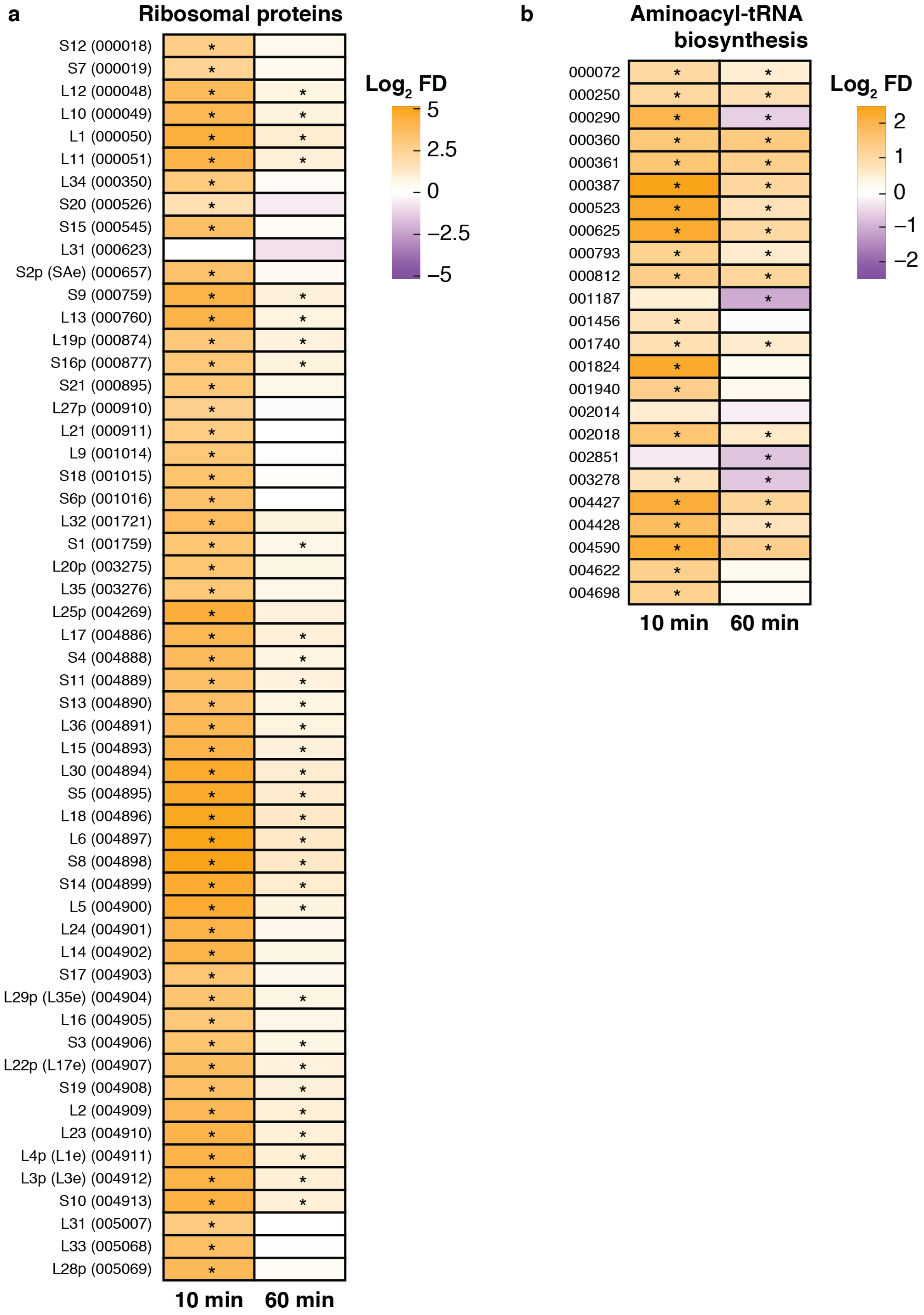
a
Ribosomal proteins
S12 (000018)
S7 (000019)
L12 (000048)
L10 (000049)
L1 (000050)
L11 (000051)
L34 (000350)
S20 (000526)
S15 (000545)
L31 (000623)
S2p (SAe) (000657)
S9 (000759)
L13 (000760)
L19p (000874)
S16p (000877)
S21 (000895)
L27p (000910)
L21 (000911)
L9 (001014)
S18 (001015)
S6p (001016)
L32 (001721)
S1 (001759)
L20p (003275)
L35 (003276)
L25p (004269)
L17 (004886)
S4 (004888)
S11 (004889)
S13 (004890)
L36 (004891)
L15 (004893)
L30 (004894)
S5 (004895)
L18 (004896)
L6 (004897)
S8 (004898)
S14 (004899)
L5 (004900)
L24 (004901)
L14 (004902)
S17 (004903)
L29p (L35e) (004904)
L16 (004905)
S3 (004906)
L22p (L17e) (004907)
S19 (004908)
L2 (004909)
L23 (004910)
L4p (L1e) (004911)
L3p (L3e) (004912)
S10 (004913)
L31 (005007)
L33 (005068)
L28p (005069)
10 min
60 min
Log2 FD
5
2.5
0
−2.5
−5
b
Aminoacyl-tRNA biosynthesis
000072
000250
000290
000360
000361
000387
000523
000625
000793
000812
001187
001456
001740
001824
001940
002014
002018
002851
003278
004427
004428
004590
004622
004698
10 min
60 min
Log2 FD
2
1
0
−1
−2

Supplementary Figure 11 | Heatmaps of ribosomal protein and aminoacyl-tRNA biosynthesis gene expression. Log_2-transformed fold differences between mucus and control (log_2 FD) and their adjusted *p* values were determined by using DESeq2. Columns represent time points, colors indicate log_2 FD, and asterisks mark genes with significant differential expression (adjusted $p < 0.05$). Numbers in row labels represent NCBI locus tags, and follow the prefix, "VIC_". Ribosomal protein (**a**) and aminoacyl-tRNA biosynthesis genes (**b**) were identified through KEGG 03010 and KEGG 00970 pathway gene assignments, respectively.

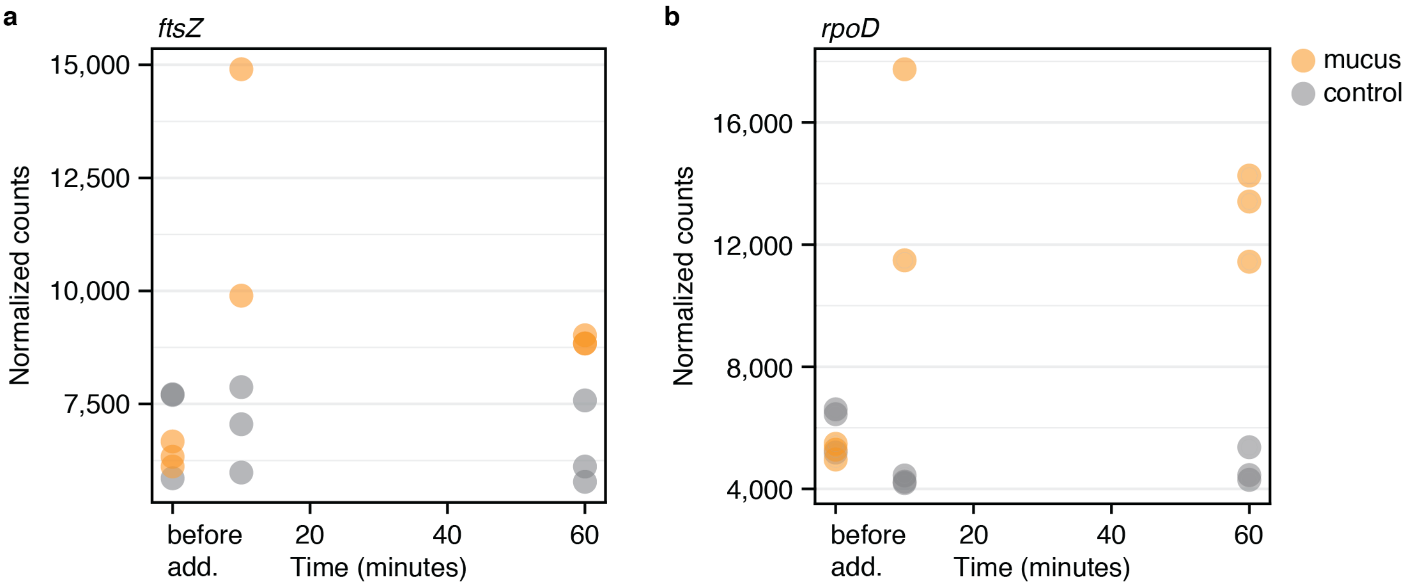

Supplementary Figure 12 | Growth-related genes were upregulated in coral mucus. Raw transcript counts were normalized by library size ("Normalized counts"). Each data point represents a sample sequenced by RNA-seq. (**a**) FtsZ (EEX34708) is required for bacterial cell division. Differential expression analysis (DESeq2) showed that at 10 min, upregulation of *ftsZ* in mucus was significant at adjusted $p < 0.01$ (fold change mucus vs. control, 1.78×) but at 60 min, upregulation was significant only at adjusted $p < 0.05$ (1.37×). (**b**) RpoD (EEX34866) is the principal RNA polymerase sigma factor for the transcription of growth-related genes. Upregulation of *rpoD* in mucus was significant at both 10 and 60 min (adjusted $p < 0.01$; 3.41× at 10 min and 2.78× at 60 min).

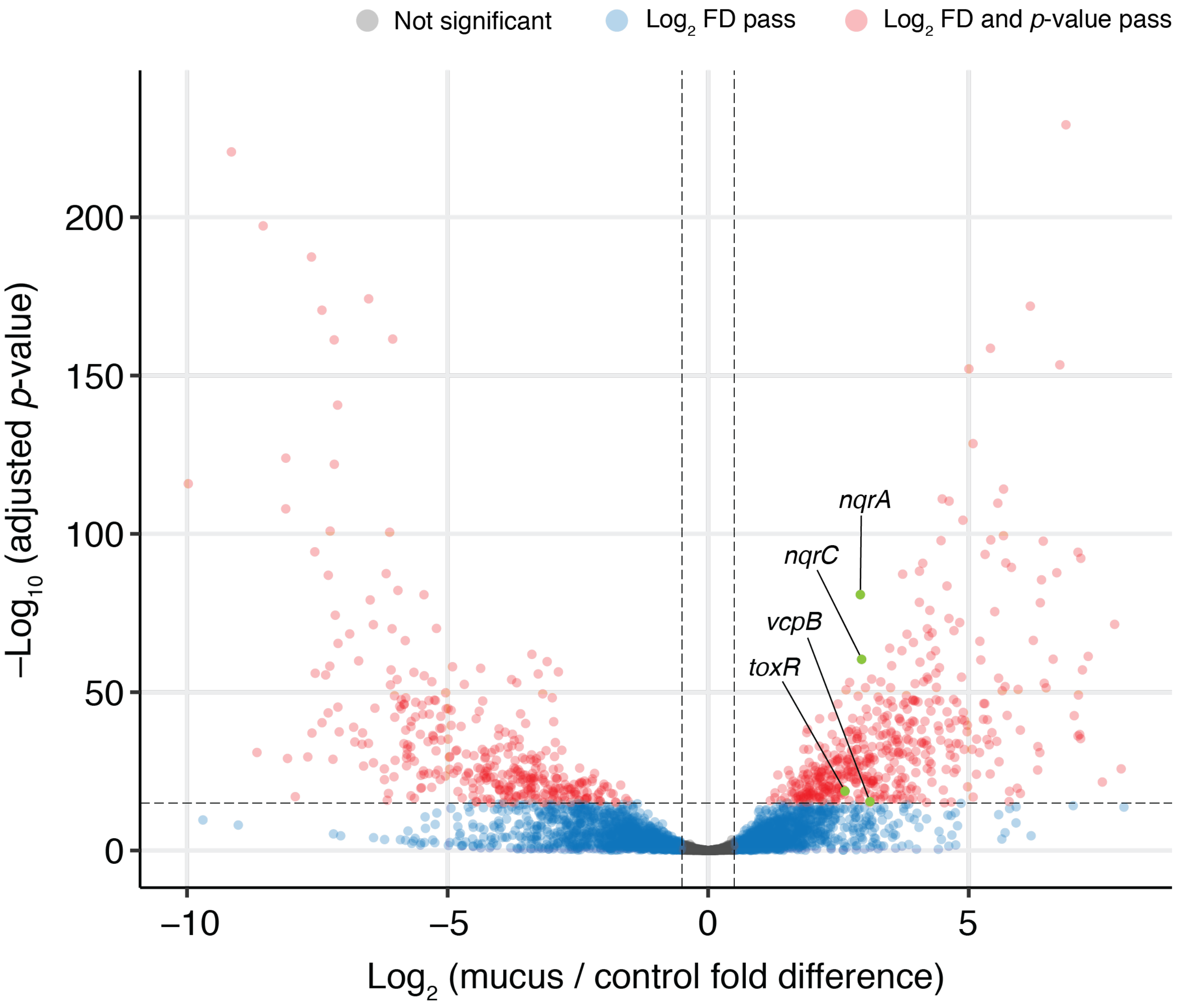

Supplementary Figure 13 | Volcano plot of differential expression at 10 minutes. Log_2-transformed fold differences between mucus and control (log_2 FD) at 10 min and their adjusted *p* values (negative log_{10}-transformed) were determined by using DESeq2. Each point represents a single gene. For visualization only, the plot is segmented by cutoffs on log_2 FD at –0.5 and 0.5, and adjusted *p* value at 1×10^{-15} (dotted lines; note that these cutoffs differ from thresholds applied in DESeq2 analyses). Four notable genes that are strongly and significantly upregulated are highlighted (green dots): NA^+-NQR genes *nqrA* (EEX34667) and *nqrC* (EEX34665); virulence-implicated zinc metalloprotease *vcpB* (EEX32371); virulence master regulator *toxR* (EEX35320).

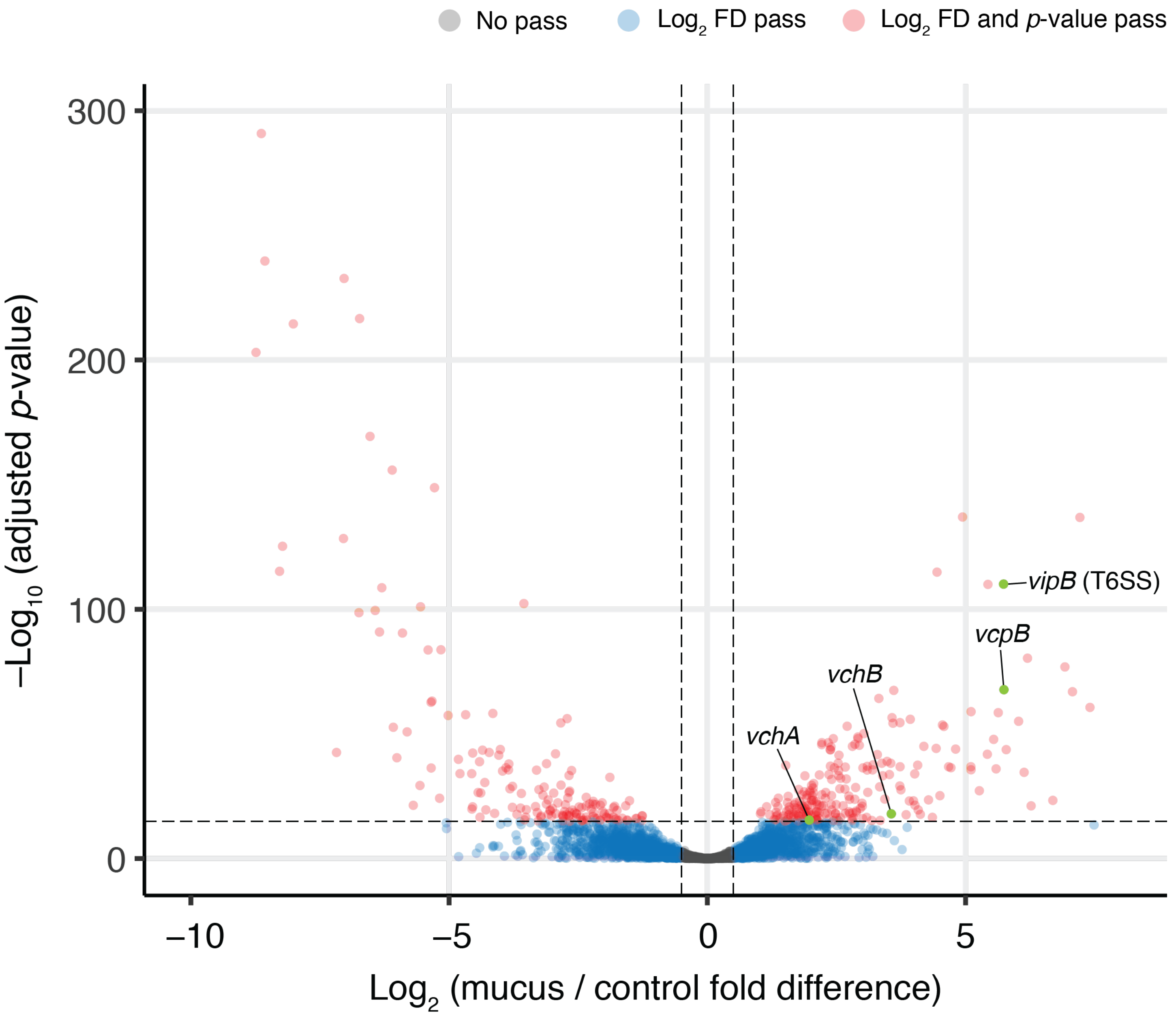

Supplementary Figure 14 | Volcano plot of differential expression at 60 minutes. Same as Supplementary Figure 13 except at the 60 min time point. Four notable genes that are strongly and significantly upregulated are highlighted (green dots): Type 6 secretion system gene *vipB* (EEX32048); virulence-implicated zinc metalloprotease *vcpB* (EEX32371); hemolysins *vchA* (EEX31069) and *vchB* (EEX31068).

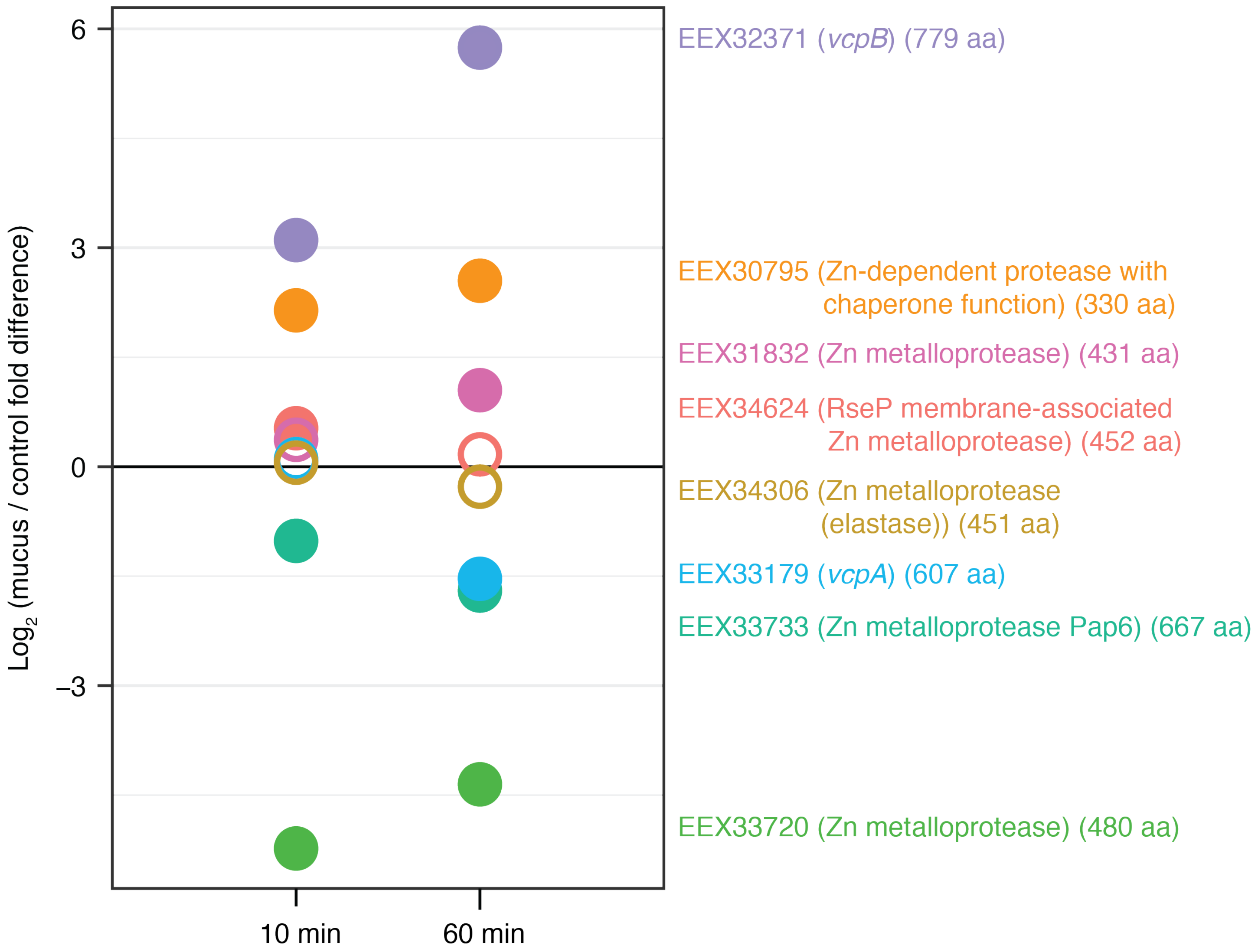

Supplementary Figure 15 | Differential expression of zinc metalloprotease genes in the *V. coralliilyticus* genome. Eight zinc metalloproteases are shown and discussed in Supplementary Discussion. Solid circles, statistically significant; open circles, not significant (adjusted $p < 0.01$).

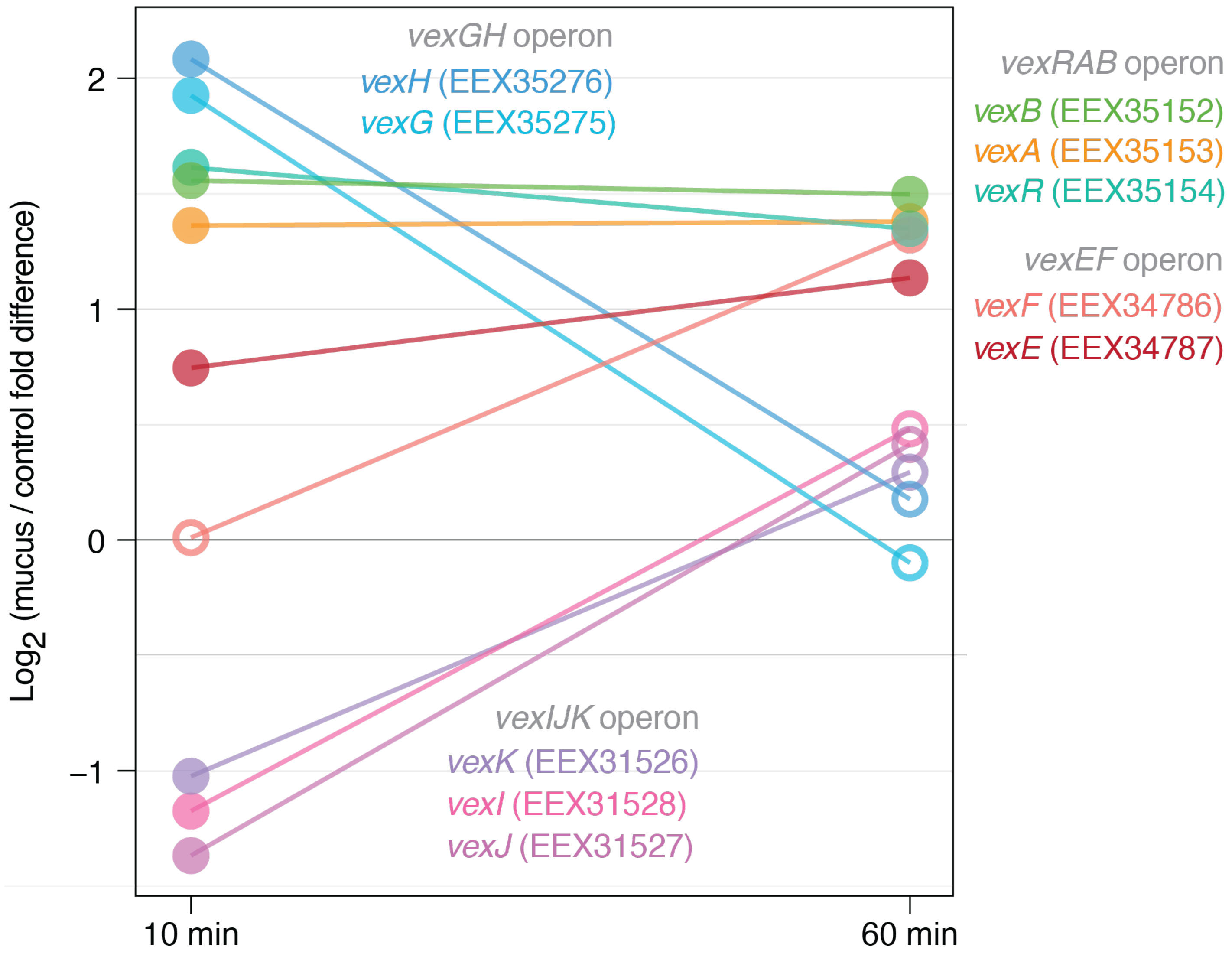

Supplementary Figure 16 | Differential expression of genes encoding RND multidrug efflux systems. The *vex* genes encoding RND multidrug efflux pumps were identified in the *V. coralliilyticus* genome through amino acid sequence homology with *vex* genes of *V. cholerae*, in which the RND efflux systems have been found to be required for antimicrobial resistance and optimal virulence factor production (11,12). Similarly to *V. cholerae*, the *vex* genes are clustered in operons (gray text) in the *V. coralliilyticus* genome. Unlike *V. cholerae*, *vexC* was equivalent to *vexI*, and *vexD* was equivalent to *vexK* in *V. coralliilyticus*. Furthermore, no homology was found for *vexLM* of *V. cholerae*. Solid circles, statistically significant; open circles, not significant (adjusted $p < 0.05$).

3. Supplementary Tables

Supplementary Table 1. Differential expression analysis results (DESeq2) of pairwise comparison, coral mucus (10 min) vs. control (10 min) (in accompanying Excel file).

Supplementary Table 2. Differential expression analysis results (DESeq2) of pairwise comparison, coral mucus (60 min) vs. control (60 min) (in accompanying Excel file).

Supplementary Table 3. Summary of differential expression analysis results (DESeq2 FDR cutoff at adjusted $p < 0.01$).

Differential expression at 10 min	Differential expression at 60 min	Number of genes (% of total genes)
Up	Up	634 (12.6%)
Down	Down	679 (13.5%)
Up	Down	118 (2.4%)
Down	Up	90 (1.8%)
**Total number of genes differentially expressed at both 10 and 60 min**		**1521 (30.3%)**

Supplementary Table 4. List of 100 *V. coralliilyticus* genes that were most significantly differentially expressed at 10 minutes. Genes are sorted first by adjusted *p* values, then by $\log_2$ fold difference from pairwise comparison of coral mucus (10 min) vs. control (10 min).

#	GenBank accession	Gene product name	$\log_2$ fold difference	Adjusted *p* value
**Upregulated in mucus**				
1	EEX33015.1	cobalt-zinc-cadmium resistance protein czcA	10.6566036	9.93E-99
2	EEX34074.1	proton/glutamate symport protein	9.16765548	2.21E-230
3	EEX33014.1	hypothetical protein	8.68655938	6.13E-124
4	EEX32322.1	maltoporin	8.51898588	1.53E-150
5	EEX30917.1	imidazolonepropionase	7.79929087	3.80E-72
6	EEX32286.1	permease	7.29195188	4.92E-62
7	EEX35011.1	carbon starvation protein A	7.18158551	9.27E-58
8	EEX30915.1	urocanate hydratase	7.15083777	5.19E-93
9	EEX30914.1	histidine ammonia-lyase	7.09646689	6.42E-95
10	EEX32326.1	serine hydroxymethyltransferase	6.86563018	6.38E-230
11	EEX32328.1	glycine dehydrogenase (decarboxylating)	6.74844777	4.20E-154
12	EEX34598.1	multidrug efflux pump component MtrF	6.68917031	1.89E-88
13	EEX34604.1	acyl-CoA dehydrogenase short-chain specific	6.61989584	3.79E-61
14	EEX30923.1	formate-dependent phosphoribosylglycinamide formyltransferase	6.42949641	1.98E-98
15	EEX31998.1	glycerol kinase	6.39580172	3.29E-86
16	EEX32016.1	putative NADH oxidase	6.37357105	5.80E-79
17	EEX35442.1	xanthine/uracil/thiamine/ascorbate permease family protein	6.23865965	4.43E-67

18	EEX32298.1	putative pullulanase precursor	6.1816164	1.19E-172
19	EEX33239.1	maltose/maltodextrin transport ATP-binding protein MalK	5.81901806	3.86E-90
**20**	**EEX32739.1**	**macrophage infectivity potentiator-related protein**	**5.71059916**	**1.57E-91**
21	EEX33580.1	hypothetical protein	5.66880306	7.23E-115
22	EEX32841.1	proton/glutamate symporter	5.66706003	3.77E-100
23	EEX33240.1	maltooligosaccharide-binding protein	5.55807483	2.08E-110
24	EEX33022.1	predicted arginine uptake transporter	5.49896441	3.68E-76
**25**	**EEX31713.1**	**methyl-accepting chemotaxis protein**	**5.42372756**	**8.43E-99**
26	EEX33579.1	cysteine desulfurase	5.41707269	2.48E-159
27	EEX33840.1	2,4-dienoyl-CoA reductase [NADPH]	5.31382424	3.08E-94
28	EEX33898.1	3-ketoacyl-CoA thiolase	5.2315967	6.56E-61
29	EEX34106.1	D-amino acid dehydrogenase small subunit	5.21387567	8.24E-67
30	EEX35229.1	amino acid ABC transporter substrate-binding protein	5.08280362	3.14E-129
31	EEX33177.1	predicted transcriptional regulator	5.0029201	7.91E-153
32	EEX33897.1	enoyl-CoA hydratase/delta(3)-cis-delta(2)-trans-enoyl-CoA isomerase/3-hydroxyacyl-CoA dehydrogenase/3-hydroxybutyryl-CoA epimerase	4.88878765	4.84E-105
33	EEX32397.1	hypothetical protein	4.82656344	1.03E-72
34	EEX33044.1	periplasmic alpha-amylase	4.71699406	3.75E-70
35	EEX31285.1	ribonucleotide reductase of class Ia (aerobic) alpha subunit	4.62330791	4.58E-111
36	EEX32329.1	hypothetical protein	4.61972112	5.25E-74
**37**	**EEX31282.1**	**methyl-accepting chemotaxis protein (Cache sensor)**	**4.5823632**	**2.89E-84**
38	EEX33702.1	alanine dehydrogenase	4.49076586	9.60E-112
39	EEX32321.1	maltose operon periplasmic protein MalM	4.46891373	1.36E-98
40	EEX31583.1	transcriptional regulator, TetR family	4.43805121	1.85E-58
41	EEX30617.1	SSU ribosomal protein S10P	4.3703164	9.46E-57
42	EEX31837.1	xanthine/uracil permease	4.36342242	8.08E-64
43	EEX35227.1	amino acid ABC transporter ATP-binding protein	4.2975494	1.69E-69
44	EEX32241.1	glycosidase	4.27467001	3.27E-62
45	EEX33241.1	maltooligosaccharide ABC transporter membrane protein	4.25343375	1.45E-76
**46**	**EEX33475.1**	**methyl-accepting chemotaxis protein (Pas/Pac sensor)**	**4.23252507**	**1.94E-68**
47	EEX33830.1	sulfate permease	4.2055486	9.29E-71
48	EEX32327.1	glycine cleavage system H protein	4.1178951	1.96E-91
49	EEX34056.1	phosphoribosylformylglycinamidine cyclo-ligase	4.06505349	3.35E-60
50	EEX33555.1	L-serine dehydratase	4.0548527	6.14E-89
51	EEX32740.1	l-2-haloalkanoic acid dehalogenase HAD superfamily protein	4.0533089	4.22E-79
52	EEX33936.1	amidophosphoribosyltransferase	3.93862721	2.00E-66
53	EEX34013.1	[ThiS-adenylate] sulfurtransferase	3.81586261	5.02E-69
54	EEX31286.1	ribonucleotide reductase of class Ia (aerobic) beta subunit	3.73231893	5.36E-88
55	EEX33832.1	long-chain-fatty-acid--CoA ligase	3.71761202	8.06E-64
56	EEX35268.1	5-(carboxyamino)imidazole ribonucleotide synthase	3.52135049	4.42E-59
57	EEX32044.1	transcriptional regulator, AsnC family	3.48360993	1.33E-64
**58**	**EEX34665.1**	**Na(+)-translocating NADH-quinone reductase subunit C (NqrC)**	**2.94495236**	**4.19E-61**
**59**	**EEX34667.1**	**Na(+)-translocating NADH-quinone reductase subunit A (NqrA)**	**2.92008554**	**1.53E-81**
**Downregulated in mucus**				
1	EEX34882.1	putative hemolysin	-11.224845	6.40E-136
2	EEX35096.1	glycosyltransferase	-10.032939	1.41E-194
3	EEX35097.1	membrane-associated phospholipid phosphatase	-9.9752612	1.46E-116

4	EEX32760.1	putative phosphatase	-9.1475814	2.33E-221
5	EEX33354.1	ABC transporter, periplasmic substrate-binding protein	-8.5387418	5.39E-198
6	EEX33158.1	hypothetical protein	-8.1050309	1.24E-108
7	EEX33200.1	UDP-glucose dehydrogenase	-8.1036764	1.20E-124
**8**	**EEX32441.1**	**phosphate ABC transporter substrate-binding protein (PstS)**	**-7.6113156**	**3.60E-188**
9	EEX33364.1	transcriptional regulator	-7.5476702	4.75E-95
**10**	**EEX30802.1**	**PhoH-like ATPase**	**-7.5397774**	**1.04E-56**
11	EEX32754.1	phosphoglycolate phosphatase	-7.4101961	2.29E-171
12	EEX32881.1	C4-dicarboxylate-binding protein	-7.3397322	3.57E-56
13	EEX32017.1	alkaline phosphatase	-7.2886016	1.18E-87
14	EEX32259.1	phosphoserine phosphatase	-7.2600894	5.83E-59
15	EEX32752.1	carbohydrate ABC transporter substrate-binding protein	-7.2532991	1.30E-101
**16**	**EEX31192.1**	**methyl-accepting chemotaxis protein**	**-7.1755851**	**5.50E-162**
**17**	**EEX34149.1**	**phosphate regulon transcriptional regulatory protein (PhoB)**	**-7.1733708**	**1.07E-122**
18	EEX32818.1	hypothetical protein	-7.1551303	4.86E-75
19	EEX32755.1	carbohydrate ABC transporter ATP-binding protein	-7.1102302	2.17E-141
20	EEX32937.1	microbial collagenase secreted	-7.1018711	4.00E-66
21	EEX34285.1	hypothetical protein	-6.8779039	3.88E-69
22	EEX32753.1	putative secreted protein	-6.7062364	1.31E-60
**23**	**EEX32440.1**	**phosphate ABC transporter membrane protein 1 (PstC)**	**-6.5141418**	**6.01E-175**
**24**	**EEX34148.1**	**phosphate regulon sensor protein (PhoR)**	**-6.4849994**	**7.43E-80**
25	EEX31160.1	staphylolysin. Metallo peptidase. MEROPS family M23A	-6.4240748	4.73E-72
26	EEX32765.1	hypothetical protein	-6.1787542	3.61E-88
**27**	**EEX32439.1**	**phosphate ABC transporter membrane protein 2 (PstA)**	**-6.1117239**	**3.05E-101**
**28**	**EEX32726.1**	**chemotaxis protein CheA**	**-6.0838184**	**9.27E-58**
29	EEX33717.1	glutamate decarboxylase	-6.0650682	9.31E-71
**30**	**EEX32438.1**	**phosphate ABC transporter ATP-binding protein (PstB)**	**-6.0524215**	**3.00E-162**
31	EEX31686.1	alkaline phosphatase	-5.9527367	7.27E-83
**32**	**EEX33718.1**	**methyl-accepting chemotaxis protein (Cache sensor)**	**-5.8120389**	**5.17E-67**
33	EEX33725.1	hypothetical protein	-5.6464007	5.42E-57
34	EEX32024.1	hypothetical protein	-5.4533219	1.64E-81
**35**	**EEX32733.1**	**Che cluster related two-component response regulator**	**-5.2133035**	**7.29E-71**
**36**	**EEX33217.1**	**phosphate ABC transporter substrate-binding protein**	**-4.9045433**	**9.26E-59**
37	EEX33095.1	hypothetical protein	-4.3650866	2.90E-58
38	EEX35279.1	hypothetical protein	-3.3823566	1.13E-62
39	EEX35185.1	transcriptional regulator AraC/XylS family	-3.2612591	1.76E-56
40	EEX35184.1	threonine efflux protein	-3.0892819	2.20E-60
41	EEX35194.1	hypothetical protein	-2.8734852	3.85E-57

Red Putative virulence-related genes
Bold Chemotaxis-related genes
Purple Na^+-NQR enzyme genes
Green Phosphate regulation genes with putative connections to virulence

Supplementary Table 5. List of 100 *V. coralliilyticus* genes that were most significantly differentially expressed at 60 minutes. Genes are sorted first by adjusted *p* values, then by $\log_2$ fold difference from pairwise comparison of coral mucus (60 min) vs. control (60 min).

#	GenBank accession	Gene product name	Log_2 fold difference	Adjusted *p* value
**Upregulated in mucus**				
1	EEX31999.1	glycerol uptake facilitator protein	7.40848654	2.30E-61
2	EEX31998.1	glycerol kinase	7.21332462	1.44E-137
3	EEX35090.1	glycerol-3-phosphate transporter	7.06957256	1.19E-67
4	EEX31989.1	homodimeric glycerol 3-phosphate dehydrogenase (quinone)	6.92333158	1.33E-77
5	EEX33444.1	hypothetical protein	6.19910525	4.23E-81
6	EEX32058.1	ClpB protein	6.02673103	8.52E-56
7	EEX33015.1	cobalt-zinc-cadmium resistance protein czcA	5.78661754	1.98E-44
**8**	**EEX32371.1**	**zinc metalloprotease (VcpB)**	**5.74273607**	**1.78E-68**
**9**	**EEX32048.1**	**uncharacterized protein ImpC (VipB of T6SS)**	**5.73561841**	**7.84E-111**
**10**	**EEX32353.1**	**protease**	**5.63236912**	**2.96E-59**
**11**	**EEX32019.1**	**metalloprotease**	**5.54347655**	**1.55E-48**
**12**	**EEX34099.1**	**alkaline serine protease**	**5.43283505**	**1.04E-110**
**13**	**EEX32370.1**	**aminopeptidase**	**5.421236**	**1.43E-42**
14	EEX33014.1	hypothetical protein	5.10638813	1.16E-59
15	EEX32041.1	hypothetical protein	5.10265631	1.46E-37
16	EEX33885.1	UPF0061 domain-containing protein	4.94128021	9.81E-138
17	EEX31899.1	Phosphocarrier protein HPr/PTS system D-fructose-specific IIA component	4.80734402	1.19E-44
18	EEX33901.1	long-chain fatty acid transport protein	4.71619906	3.62E-37
19	EEX31898.1	1-phosphofructokinase	4.67180086	1.48E-37
20	EEX32000.1	glycerol 3-phosphate dehydrogenase (quinone) subunit A	4.58023526	1.00E-53
21	EEX32322.1	maltoporin	4.55237858	2.52E-54
22	EEX32298.1	putative pullulanase precursor	4.44666553	1.26E-115
23	EEX31648.1	aldehyde dehydrogenase	4.43044939	6.39E-45
24	EEX32113.1	betaine aldehyde dehydrogenase	4.1917458	9.39E-46
**25**	**EEX33113.1**	**aminopeptidase Y (Arg Lys Leu preference)**	**4.07209529**	**3.44E-38**
26	EEX34954.1	aspartate kinase	3.92897031	1.36E-56
27	EEX32114.1	transcriptional regulator, TetR family	3.73131125	1.45E-37
28	EEX32112.1	choline dehydrogenase	3.72914504	2.89E-55
29	EEX31647.1	choline dehydrogenase	3.60929868	3.43E-68
30	EEX35321.1	chaperone protein HtpG	3.59435654	5.01E-55
31	EEX32471.1	putative periplasmic substrate-binding protein	3.5756782	3.00E-57
32	EEX30735.1	hypothetical protein	3.55109177	5.81E-39
33	EEX30720.1	heat shock protein 60 family co-chaperone GroES	3.48014351	1.08E-39
34	EEX32472.1	L-proline Glycine Betaine ABC transport system permease proW	3.37742632	5.84E-39
35	EEX32109.1	L-proline Glycine Betaine ABC transport ATP-binding protein proV	3.32015589	5.70E-65
36	EEX34073.1	chaperone protein DnaK	3.29161547	1.99E-42
**37**	**EEX32739.1**	**macrophage infectivity potentiator-related protein**	**3.21027974**	**2.44E-38**
38	EEX30705.1	ATP-dependent hsl protease ATP-binding subunit HslU	3.09672866	9.94E-38
39	EEX30719.1	heat shock protein 60 family chaperone GroEL	3.03216849	7.15E-51
**40**	**EEX35183.1**	**gamma-glutamyltranspeptidase**	**2.94236444**	**3.65E-46**

41	EEX34072.1	chaperone protein DnaJ	2.91701728	7.56E-49
**42**	**EEX34157.1**	**protein-export membrane protein SecD**	**2.91377221**	**1.74E-49**
43	EEX32202.1	pentapeptide repeat family protein	2.85785441	1.46E-46
44	EEX35091.1	glycerophosphoryl diester phosphodiesterase	2.80854053	9.48E-46
45	EEX32044.1	transcriptional regulator, AsnC family	2.70701783	8.23E-54
46	EEX30796.1	membrane protein	2.67090846	1.98E-37
**47**	**EEX30795.1**	**Zn-dependent protease with chaperone function**	**2.54844484**	**3.50E-42**
48	EEX33871.1	cell division trigger factor	2.53288819	5.51E-39
49	EEX35368.1	type III restriction enzyme res subunit	2.43867985	7.82E-49
50	EEX32473.1	L-proline Glycine Betaine ABC transport ATP-binding protein proV	2.39323275	1.50E-45
**51**	**EEX34158.1**	**protein translocase subunit yajC**	**2.38450408**	**2.27E-44**
**52**	**EEX34156.1**	**protein translocase subunit secF**	**2.38285125**	**8.39E-38**
**53**	**EEX30690.1**	**protein translocase subunit secB**	**2.37785509**	**2.27E-44**
**54**	**EEX34706.1**	**protein translocase subunit secA**	**2.34129002**	**3.28E-47**
55	EEX30698.1	YjbH outer membrane lipoprotein	2.21671352	3.28E-47
56	EEX33177.1	predicted transcriptional regulator	2.21413958	1.87E-46
57	EEX32908.1	chromosome segregation ATPase	1.51818514	4.12E-38
**Downregulated in mucus**				
1	EEX33074.1	hypothetical protein	-2.7149682	7.23E-57
2	EEX35185.1	transcriptional regulator AraC/XylS family	-2.837767	3.88E-55
3	EEX33357.1	ABC transporter, permease protein	-2.9392167	9.41E-43
4	EEX33073.1	multidrug resistance protein D	-3.1138029	8.46E-39
5	EEX35184.1	threonine efflux protein	-3.5521563	4.99E-103
6	EEX34475.1	hypothetical protein	-3.8430412	1.12E-38
**7**	**EEX34148.1**	**phosphate regulon sensor protein (PhoR)**	**-4.0082037**	**1.74E-44**
8	EEX30987.1	transcriptional regulator, GntR family	-4.0384179	3.46E-42
9	EEX31335.1	hypothetical protein	-4.1526739	6.00E-59
10	EEX33356.1	ABC transporter, ATP-binding protein	-4.2238716	2.72E-43
**11**	**EEX33720.1**	**zinc metalloprotease**	**-4.3527684**	**3.26E-44**
12	EEX32753.1	putative secreted protein	-4.4104483	1.38E-39
13	EEX32259.1	phosphoserine phosphatase	-4.5430871	4.58E-43
14	EEX32818.1	hypothetical protein	-4.6800137	1.97E-58
15	EEX32751.1	carbohydrate ABC transporter membrane protein 2, CUT1 family	-4.8199352	1.57E-40
16	EEX30988.1	methylisocitrate lyase	-5.0190929	3.98E-58
**17**	**EEX34149.1**	**phosphate regulon transcriptional regulatory protein (PhoB)**	**-5.1578667**	**1.76E-84**
**18**	**EEX32438.1**	**phosphate ABC transporter ATP-binding protein (PstB)**	**-5.2829748**	**1.71E-149**
19	EEX30990.1	2-methylcitrate dehydratase FeS dependent	-5.3310035	6.77E-64
20	EEX30991.1	2-methylaconitate cis-trans isomerase	-5.3501319	2.03E-63
21	EEX30989.1	2-methylcitrate synthase	-5.4068002	2.09E-84
**22**	**EEX32439.1**	**phosphate ABC transporter membrane protein 2 (PstA)**	**-5.5524533**	**1.01E-101**
23	EEX33360.1	hypothetical protein	-5.813381	1.50E-51
24	EEX32752.1	carbohydrate ABC transporter substrate-binding protein, CUT1 family	-5.9032963	3.41E-91
25	EEX32750.1	carbohydrate ABC transporter membrane protein 1, CUT1 family	-6.011247	3.59E-41
26	EEX30992.1	propionyl-CoA synthetase	-6.0804617	2.11E-53
27	EEX32755.1	carbohydrate ABC transporter ATP-binding protein, CUT1 family	-6.1019383	1.50E-156
28	EEX33200.1	UDP-glucose dehydrogenase	-6.3025751	2.30E-109
29	EEX31160.1	staphylolysin. Metallo peptidase. MEROPS family M23A	-6.3471964	1.29E-91

**30**	**EEX33217.1**	**phosphate ABC transporter substrate-binding protein**	**-6.431385**	**2.99E-100**
**31**	**EEX31192.1**	**methyl-accepting chemotaxis protein**	**-6.5310413**	**4.50E-170**
32	EEX32754.1	phosphoglycolate phosphatase	-6.7327592	2.47E-217
33	EEX33158.1	hypothetical protein	-6.7454801	2.28E-99
**34**	**EEX32440.1**	**phosphate ABC transporter membrane protein 1 (PstC)**	**-7.0333295**	**2.01E-233**
35	EEX33364.1	transcriptional regulator	-7.0455797	4.15E-129
36	EEX31630.1	hypothetical protein	-7.179953	2.75E-43
37	EEX33354.1	ABC transporter, periplasmic substrate-binding protein	-8.0163816	3.34E-215
38	EEX35097.1	membrane-associated phospholipid phosphatase	-8.2248773	5.37E-126
39	EEX32017.1	alkaline phosphatase	-8.2823799	5.81E-116
40	EEX32760.1	putative phosphatase	-8.5664181	2.03E-240
**41**	**EEX32441.1**	**phosphate ABC transporter substrate-binding protein (PstS)**	**-8.6359639**	**1.47E-291**
42	EEX35096.1	glycosyltransferase	-8.7388328	9.47E-204
43	EEX34882.1	putative hemolysin	-10.711944	2.70E-158

Red Putative virulence-related genes, including secretion systems
Orange Proteases
Bold Chemotaxis-related genes
Green Phosphate regulation genes with putative connections to virulence

Supplementary Table 6. Na^+-NQR genes shown in Fig. 4a.

Gene	*V. alginolyticus* gene*	*V. coralliilyticus* gene	*V. coralliilyticus* gene locus	Identity (%)	Coverage (%)
nqrA	GAD71943	EEX34667	VIC_000693	92.52	89
nqrB **	GAD71712	EEX34666	VIC_000692	67.48	99
nqrC	GAD71945	EEX34665	VIC_000691	80.08	100
nqrD	GAD71946	EEX34664	VIC_000690	98.57	100
nqrE	GAD71947	EEX34663	VIC_000689	98.48	100
nqrF	GAD71948	EEX34662	VIC_000688	97.05	100

* *nqr* genes were identified in the *V. coralliilyticus* genome through high amino acid homology (queried on BLASTp) with *nqr* genes of *V. alginolyticus* (13).

** Two genes with high homology to *nqrB* was found in the *V. coralliilyticus* genome (EEX32277, not shown). Only the gene (EEX34666) that is adjacent to the other *nqr* genes is shown.

Supplementary Table 7. Biofilm (*vps* and *rbm*) genes shown in Fig. 4b.

Gene	*V. cholerae* gene*	*V. coralliilyticus* gene	*V. coralliilyticus* gene locus	Identity (%)	Coverage (%)
vpsR	AWB73202	EEX35032	VIC_000540	86.39	99
vpsU	AWK28764	EEX33051	VIC_002501	43.57	83
vpsA	AWB73428	EEX33052	VIC_002502	77.09	99
vpsB	AWB73429	EEX33053	VIC_002503	83.05	100
vpsC	AWB73430	-	-	-	-
vpsD	AWB73431	EEX33054	VIC_002504	37.31	99
vpsE	AWB73432	EEX33055	VIC_002505	36.23	99
vpsF	AWB73433	EEX33056	VIC_002506	46.41	89
vpsG	AWB73434	EEX33057	VIC_002507	41.22	91
vpsH	AWB73435	EEX33058	VIC_002508	57.76	93
vpsI	AWB73436	EEX33059	VIC_002509	54.49	99
vpsJ	AWB73437	EEX33060	VIC_002510	58.95	97
vpsK	AWB73438	EEX33061	VIC_002511	77.59	98
vpsT	AWB76290	EEX33067	VIC_002517	57.28	91
vpsL	AWB73444	EEX33156	VIC_002610	77.16	100
vpsM	AWB73445	EEX33155	VIC_002609	56.85	98
vpsN	AWB73446	EEX33154	VIC_002608	67.82	99
vpsO	AWB73447	EEX33153	VIC_002607	68.18	98
vpsP	AWB73448	EEX33152	VIC_002606	31.28	88
vpsQ	AWB73449	-	-	-	-
rbmA	AAF94090 (VC_0928)	EEX33062	VIC_002512	41.28	85
rbmB	AAF94091 (VC_0929)	EEX33063	VIC_002513	47.62	92
rbmC / *bap1*	AAF94092 (VC_0930)	EEX33064	VIC_002514	66.42	99
rbmD	AAF94093 (VC_0931)	EEX33150	VIC_002604	59.29	97
rbmE	AAF94094 (VC_0932)	EEX33151	VIC_002605	67.86	88
rbmF	AAF94095 (VC_0933)	-	-	-	-

* *vps* and *rbm* genes were identified in the *V. coralliilyticus* genome through high amino acid homology (queried on BLASTp) with *vpsA–Q, vpsR, vpsT* and *rbmABCDEF* genes of *V. cholerae* (14,15). Additional information is available in Supplementary Discussion.

Supplementary Table 8. Quorum sensing, transcription regulator, and toxin genes shown in Fig. 4c–e.

Gene	*V. coralliilyticus* gene	Homologous genes in other Vibrios	References
**Quorum sensing autoinducer synthases**			
luxM (AI-1 synthase)	EEX31502	*luxM*	(10)
luxS (AI-2 synthase)	EEX35562	*luxS*	(10)
cqsA (CAI-1 synthase)	EEX33462	*cqsA*	(10)
**Master transcription regulators**			
toxR	EEX35320	*toxR*	(16–19)
toxS	EEX35319	*toxS*	(20)
aphA	EEX30687	*aphA*	(21)
vcpR	EEX34823	*hapR, luxR*	(22,23)
**Toxins**			
vcpA	EEX33179	*hapA*	(7,10,22,24–26)
vcpB	EEX32371	*hapA*	(7,10,22,26)
vchA	EEX31069	*vvhA*	(10,22)
vchB	EEX31068	*vvhB*	(10,22)

Supplementary Table 9. Zinc metalloprotease genes shown in Supplementary Figure 15 and discussed in Supplementary Discussion.

***V. coralliilyticus* gene**	***V. coralliilyticus* locus tags**	**Gene name**	**Length (aa)**	**Identity (%) HapA***	**References**
EEX33179	VIC_002633	*vcpA*	607	69.41	(7,10,22,24,25)
EEX32371	VIC_003472	*vcpB*	779	49.11	(7,10,22,26)
EEX33720	VIC_001616	Zinc metalloprotease	480	48.42	NCBI annotation
EEX33733	VIC_001629	Zinc metalloprotease Pap6	667	41.65	NCBI annotation
EEX31832	VIC_004782	Zinc metalloprotease	431	39.13	NCBI annotation
EEX34624	VIC_000650	Membrane-associated zinc metalloprotease (RseP)	452	33.33	NCBI annotation
EEX30795	VIC_004815	Zn-dependent protease with chaperone function (YfgC)	330	33.33	NCBI annotation
EEX34306	VIC_001102	Zinc metalloprotease (elastase)	451	28.30	NCBI annotation

* Amino acid (aa) sequence identity with *V. cholerae* HapA gene (AVK79064).

Supplementary Table 10. Secretion system genes shown in Fig. 4f.

Gene	*V. coralliilyticus* gene locus*	KO number
**Type 1 Secretion System**		
tolC	VIC_000771	K12340 tolC; outer membrane protein
**Type 2 Secretion System**		
gspC	VIC_000133	K02452 gspC; general secretion pathway protein C
gspD	VIC_000132	K02453 gspD; general secretion pathway protein D
gspE	VIC_000131	K02454 gspE; general secretion pathway protein E
gspF	VIC_000130	K02455 gspF; general secretion pathway protein F
gspG	VIC_000129	K02456 gspG; general secretion pathway protein G
gspH	VIC_000128	K02457 gspH; general secretion pathway protein H
gspI	VIC_000127	K02458 gspI; general secretion pathway protein I
gspJ	VIC_000126	K02459 gspJ; general secretion pathway protein J
gspK	VIC_000125	K02460 gspK; general secretion pathway protein K
gspL	VIC_000124	K02461 gspL; general secretion pathway protein L
gspM	VIC_000123	K02462 gspM; general secretion pathway protein M
**Type 3 Secretion System (located on Coralliilyticus Pathogenicity Island-1)**		
yscR-1	VIC_004064	K03226 yscR; type III secretion protein R
yscS-1	VIC_004063	K03227 yscS; type III secretion protein S
yscT-1	VIC_004062	K03228 yscT; type III secretion protein T
yscU-1	VIC_004061	K03229 yscU; type III secretion protein U
yscC-1	VIC_004057	K03219 yscC; type III secretion protein C
yscF	VIC_004032	K03221 yscF; type III secretion protein F
yscJ-1	VIC_004029	K03222 yscJ; type III secretion protein J
yscV-1	VIC_004022	K03230 yscV; type III secretion protein V
yscN-1	VIC_004021	K03224 yscN; ATP synthase in type III secretion protein N
**Type 3 Secretion System (located on Megaplasmid)**		
yscJ-2	VIC_001055	K03222 yscJ; type III secretion protein J
yscN-2	VIC_001050	K03224 yscN; ATP synthase in type III secretion protein N
yscR-2	VIC_001046	K03226 yscR; type III secretion protein R
yscS-2	VIC_001045	K03227 yscS; type III secretion protein S
yscT-2	VIC_001044	K03228 yscT; type III secretion protein T
yscU-2	VIC_001043	K03229 yscU; type III secretion protein U
yscV-2	VIC_001039	K03230 yscV; type III secretion protein V
yscC-2	VIC_001022	K03219 yscC; type III secretion protein C
**Type 6 Secretion System**		
vasH	VIC_003158	-
hcp	VIC_003156	-
vgrG	VIC_003155	-
vipA	VIC_003148	-
vipB	VIC_003147	-
vasA	VIC_003145	-
vasK	VIC_003142	-

vasF	VIC_003136	-
**Sec Secretion System**		
secA	VIC_000732	K03070 secA; preprotein translocase subunit SecA
secB	VIC_004986	K03071 secB; preprotein translocase subunit SecB
secD-1	VIC_002055	K03072 secD; preprotein translocase subunit SecD
secF-1	VIC_002054	K03074 secF; preprotein translocase subunit SecF
yajC	VIC_002056	K03210 yajC; preprotein translocase subunit YajC
secD-2	VIC_002788	K03072 secD; preprotein translocase subunit SecD
secF-2	VIC_002789	K03074 secF; preprotein translocase subunit SecF
secY	VIC_004892	K03076 secY; preprotein translocase subunit SecY
secE	VIC_000053	K03073 secE; preprotein translocase subunit SecE
secG	VIC_000786	K03075 secG; preprotein translocase subunit SecG
ffh (SRP54)	VIC_000878	K03106 SRP54; signal recognition particle subunit SRP54
ftsY	VIC_000196	K03110 ftsY; fused signal recognition particle receptor
**Tat Secretion system**		
tatA	VIC_000431	K03116 tatA; sec-independent protein translocase protein TatA
tatB	VIC_000432	K03117 tatB; sec-independent protein translocase protein TatB
tatC	VIC_000433	K03118 tatC; sec-independent protein translocase protein TatC

* List of genes in types 1, 2, 3, Sec, and Tat secretion systems were obtained from the Bacterial Secretion System KEGG 03070 pathway gene assignment. Type 6 secretion genes were obtained from Guillemette, Ushijima et al. (27).

4. Supplementary Discussion

4.1. Investigation of swimming speed enhancement by control cells

We observed a weak and short-lived increase in swimming speed in *V. coralliilyticus* control cells that were exposed to the addition of filtered spent medium in place of coral mucus. This unexpected enhancement of swimming speed in control cells peaked at 66.3 ± 0.9 μm/s after 8 minutes, representing 1.3× the pre-addition speed (Fig. 2e). Swimming speeds returned to baseline within 15 minutes (two-tailed *t*-test with control speed at $t = -2$ min, $p > 0.01$).

This speed enhancement in filtered spent medium (also referred to as "filtrate") was observed consistently across experiments and regardless of the filter material used (cellulose acetate or polyvinylidene fluoride), the amount of pressure applied (2.8 cm^2 or 10 cm^2 filtration area) during filtrate production, or the use of slightly warmer (+1 °C) filtrate (Supplementary Fig. 5), indicating that chemicals leaching from the filter membranes, bursting of cells due to high pressure, or a small elevation in temperature upon filtrate addition are unlikely to be the cause of the increase in swimming speed in control cells. Furthermore, vigorous and repeated pipetting of cells did not cause any speed enhancement, suggesting that hydrodynamic stimuli, specifically shear forces, were not responsible for the changes in swimming speed in control cells (Supplementary Fig. 5a). In contrast, separate experiments in which we probed the addition of filtered artificial sea water (FASW) to *V. coralliilyticus* caused a speed increase of 2.4-fold (marine broth caused a 3.4-fold speed enhancement in the same experiment; Supplementary Fig. 5b), leading us to hypothesize that chemokinesis in control cells was partially caused by oxygenation of the spent medium during filtration. Indeed, oxygen has been shown to affect swimming speeds of *V. coralliilyticus*, with faster swimming speeds recorded under oxic (~65 μm/s) compared to anoxic conditions (~25 μm/s) (28).

Importantly, the speed enhancement caused by coral mucus was higher in magnitude and longer in duration than that observed in control cells (Fig. 2e). Furthermore, the addition of filtered spent medium seems to induce speed enhancement only in a subset of the control population, observed as the mere flattening of the speed distribution histogram, compared to the whole-population shift towards faster swimming speeds observed in coral mucus-exposed populations (Supplementary Fig. 6).

4.2. Gene expression patterns that point to virulence

4.2.1. Release from phosphate starvation

Release from phosphate limitation upon coral mucus addition was likely responsible for the strong and highly statistically significant downregulation of the two-component system PhoBR, and the associated ABC-type phosphate transporter, Pst at both 10 and 60 min (Supplementary Tables 4 and 5). Inactivation of the PhoBR system has been connected to the induction of biofilm formation and virulence gene expression in *V. cholerae* (29–31). Thus, the upregulation of biofilm and virulence

genes observed in our RNA-seq results (Fig. 4) may have been the result of the strong downregulation of PhoBR and Pst systems in coral mucus.

4.2.2. Evidence for *V. coralliilyticus* growth and physiological transformation in coral mucus

In addition to elevated metabolism, gene sets involved in protein production, cell membrane generation, cell growth, and energy production were significantly upregulated in coral mucus at 10 min (Fig. 3c). Almost all ribosomal protein genes (KEGG 03010) and tRNA synthetases (KEGG 00970) were significantly upregulated within 10 min of incubation with coral mucus (Fig. 3c, Supplementary Fig. 11), which suggests a rapid increase in protein production. The sustained high expression of ribosomal protein genes (KEGG 03010) at 60 min, despite the downregulation of nutrient acquisition genes (ABC transporters, KEGG 02010) in coral mucus (Fig. 3d), suggests that the bacteria may be undergoing a physiological transformation that required continued protein production, such as a transition towards host-association and virulence. Upregulation of gene sets important for cell membrane generation (fatty acid degradation (KEGG 00071) and glycerophospholipid metabolism (KEGG 00564)), and amino acid precursors for membrane fatty acids (KEGG 00280; valine, leucine, isoleucine degradation) (Fig. 3c,d) may be caused by the elevated production of cell membranes during growth. The upregulation of oxidative phosphorylation (KEGG 00190) (Fig. 3c) suggests increased energy generation as a result of incubation with coral mucus at 10 min.

4.2.3. *vps* and *rbm* (biofilm) gene expression

Homologs of *V. cholerae vps* and *rbm* biofilm-related genes were found in the *V. coralliilyticus* genome through amino acid sequence similarity (Supplementary Table 7). Similarly to the *V. cholerae* genome, the *rbmA–E* gene cluster of *V. coralliilyticus* was in the intergenic region between the two clusters *vpsU, A–K* and *vpsL–P* (Fig. 4b).

Three genes present in the *V. cholerae* genome did not lead to significant matches in the *V. coralliilyticus* genome: *vpsQ*, *vpsC*, and *rbmF* (Supplementary Table 7). VpsQ may share functional redundancy with VpsP, as mutants of *vpsP* and *vpsQ* were both found to produce biofilms similar to wildtype in *V. cholerae* (15). Similarly, *vpsC* mutants did not display compromised VPS production or biofilm formation (15), suggesting that it shares functional redundancy with other genes. Finally, *rbmE* and *rbmF* genes of *V. cholerae* share an 88-bp overlapping region and are predicted to be an operon (32), suggesting that they may serve similar functions. Thus, while *rbmF* was not found in the *V. coralliilyticus* genome, its function may be fulfilled by *rbmE*.

In the *V. cholerae* genome, genes *rbmC* (AAF94092, VC0930) and *bap1* (AAF95036, VC1888), encode secreted proteins that modulate the development of corrugated colonies, and are critical for pellicle and biofilm formation (32). An amino acid sequence homology search of the *V. cholerae* genes *bap1* and *rbmC* against the *V. coralliilyticus* genome yielded the same, single gene, EEX33064, which likely satisfy the function of both *rbmC* and *bap1* in *V. coralliilyticus* biofilms.

Homologs of both *V. cholerae* biofilm transcriptional activators, *vpsR* and *vpsT*, were found in the *V. coralliilyticus* genome (Supplementary Table 7), but while *vpsR* was significantly upregulated in coral mucus, *vpsT* was significantly downregulated. Furthermore, it has been found that *rbmA*, which is required for rugose colony formation and biofilm structure integrity in *V. cholerae*, is positively regulated by VpsR but not VpsT (33). Consistently, we observed the upregulation of both *rbmA* and its positive regulator, *vpsR*, in coral mucus (Fig. 4b). The divergent expression patterns of the biofilm transcriptional regulators *vpsR* and *vpsT* in coral mucus suggest that their activation may be temporally separated in orchestrating the multi-step process of biofilm formation.

A single *rbm* gene, *rbmB*, was significantly downregulated in coral mucus (Fig. 4b). RbmB is a putative lyase whose function may be to degrade biofilm-related polysaccharides during the development of colony corrugation and biofilm formation (32). It is possible that the function of RbmB is important at the later stages of biofilm formation, and thus is initially downregulated in coral mucus.

4.2.4. Zinc metalloproteases

Zinc metalloproteases or their homologs have been identified as key virulence factors for many *Vibrio* pathogens (34–37), including *V. coralliilyticus* during coral infection (7,24,38). In the *V. coralliilyticus* genome, several zinc metalloproteases have been identified through genomic (10,26), and biochemical or phenotypic (7,24,25,39) studies. We focused on 8 zinc metalloproteases and their expression patterns in coral mucus (Supplementary Fig. 15 and Supplementary Table 9), though other zinc metalloproteases likely exist in the *V. coralliilyticus* genome.

The most well-studied zinc metalloproteases of *V. coralliilyticus* are VcpA (EEX33179, VIC_002633) and VcpB (EEX32371, VIC_003472), which only share 48% amino acid sequence identity with each other. VcpA (69%) and VcpB (49%) share the highest homology with *V. cholerae*'s Zn-dependent hemagglutinin protease, HapA or vibriolysin (Supplementary Table 9), which is an important virulence factor attributed to multiple pathogenic activities, including degradation of mucus barriers in human intestines (34,40,41). Thus, the high amino acid sequence homology with HapA point to their possible importance in *V. coralliilyticus* pathogenicity.

VcpA is likely the zinc metalloprotease characterized by Ben-Haim et al., who observed that the protease purified from *V. coralliilyticus* BAA-450 led to tissue damage in coral tissue (*P. damicornis*) (24). VcpA was found to have 100% identify with the 17 amino acid sequence published by Ben-Haim et al. (24), while VcpB only had partial identity. VcpA was further characterized as a major virulence factor of *V. coralliilyticus* in oysters by Hasegawa et al. (25) (note that *V. tubiashii* has now been re-identified as *V. coralliilyticus*). In the present study, however, we observed a significant downregulation of the *vcpA* gene in coral mucus (Fig. 4e), despite other gene expression patterns in *V. coralliilyticus* that point to virulence. This suggests the existence of functional redundancies amongst the many zinc metalloproteases that are encoded in the *V. coralliilyticus* genome. Indeed, it has previously been found that a Δ*vcpA* mutation did not compromise coral infection by the *V. coralliilyticus* P1 strain (26), and both VcpA and VcpB were undetectable at any temperature (avirulent 24 °C or virulent 27 °C) by Kimes et al. (10). Finally, the mean nucleotide divergence of the *vcpA* gene amongst *V. coralliilyticus* isolates was found to be 3.7% compared to only 0.2% in the 16S

rRNA gene (42), pointing to the possibility that protein functions may differ even within the single species of *V. coralliilyticus*.

The second, highly studied zinc metalloprotease in *V. coralliilyticus* virulence is VcpB. The *vcpB* gene has been found to be co-regulated with *vcpA* by VcpR (shown in Fig. 4d) (22). Sussman et al. partially sequenced the putative zinc metalloprotease suspected to cause photoinactivation of coral symbionts (7). According to our amino acid sequence homology analyses of the Sussman et al. fragment sequences, both VcpA and VcpB have 100% identity with some, but mutually exclusive, fragments. Thus, it is plausible that the symbiont photoinactivation and coral tissue lesions observed by Sussman et al. was mediated by a mixture of the two zinc metalloproteases (VcpA and VcpB). Notably, *vcpB* was one of the most significantly and strongly upregulated genes in coral mucus compared to control at 60 min (Supplementary Fig. 14, Supplementary Table 5), suggesting that its function is especially important for *V. coralliilyticus* in coral mucus. Similarly, the transcription of a metalloprotease by the fish pathogen *V. anguillarum* was induced by mucus (43). Furthermore, the *V. cholerae* zinc metalloprotease HapA has been observed to degrade mucin as well as other physiological substrates (34,40,41). Thus, a more detailed investigation of this second, less studied zinc metalloprotease may yield novel insights into the mechanisms of coral infection by *V. coralliilyticus*.

The gene annotated as “Zn-dependent protease with chaperone function” (EEX30795) was also amongst the top upregulated genes with high statistical significance (Supplementary Table 5). NCBI annotation suggests that this gene may encode YfgC, which has been found to maintain integrity of the outer membrane of *E. coli* (44). Thus, it is plausible that EEX30795 may act in concert with the growth- and biofilm-related activities suggested by the gene expression patterns of *V. coralliilyticus* in coral mucus.

RseP (EEX34624) was weakly upregulated in coral mucus at 10 min but not at 60 min (Supplementary Fig. 15), and was also detected at elevated levels at the virulent temperature of 27 °C (compared to 24 °C) by Kimes et al. (10). RseP is characterized as an inner membrane-localized zinc metalloprotease that proteolyzes the master virulence regulator, ToxR, in response to nutrient limitation (45,46). Thus, the reversal of nutrient limitation upon exposure to coral mucus may have led to the return of RseP expression to baseline levels at 60 min (Supplementary Fig. 15), and as a result, the accumulation of ToxR through its upregulation is expected (Fig. 4d).

Little information is available on the three zinc metalloproteases (aside from VcpA) that were downregulated (EEX33733, EEX33720) or not differentially expressed (EEX34306) in coral mucus compared to control. These, like some zinc metalloproteases, may serve biological functions that are not virulence-related, as suggested by a previous study of coral pathogens (38).

5. Additional References

1. Xie L, Altindal T, Chattopadhyay S, Wu X-L. Bacterial flagellum as a propeller and as a rudder for efficient chemotaxis. Proc Natl Acad Sci USA. 2011;108(6):2246–51.
2. Son K, Guasto JS, Stocker R. Bacteria can exploit a flagellar buckling instability to change direction. Nat Phys. 2013;9:494–8.
3. Li H, Durbin R. Fast and accurate short read alignment with Burrows-Wheeler transform. Bioinformatics. 2009;25(14):1754–60.
4. Liao Y, Smyth GK, Shi W. featureCounts: an efficient general purpose program for assigning sequence reads to genomic features. Bioinformatics. 2014;30(7):923–30.
5. Subramanian A, Tamayo P, Mootha VK, Mukherjee S, Ebert BL, Gillette MA, et al. Gene set enrichment analysis: A knowledge-based approach for interpreting genome-wide expression profiles. Proc Natl Acad Sci USA. 2005;102(43):15545–50.
6. Mootha VK, Lindgren CM, Eriksson K-F, Subramanian A, Sihag S, Lehar J, et al. PGC-1α-responsive genes involved in oxidative phosphorylation are coordinately downregulated in human diabetes. Nat Genet. 2003;34(3):267–73.
7. Sussman M, Mieog JC, Doyle J, Victor S, Willis BL, Bourne DG. *Vibrio* zinc-metalloprotease causes photoinactivation of coral endosymbionts and coral tissue lesions. PLoS One. 2009;4(2).
8. Garren M, Son K, Raina J-B, Rusconi R, Menolascina F, Shapiro OH, et al. A bacterial pathogen uses dimethylsulfoniopropionate as a cue to target heat-stressed corals. ISME J. 2014 Dec 12;8:999–1007.
9. Kim Y-K, McCarter LL. Analysis of the polar flagellar gene system of *Vibrio parahaemolyticus*. J Bacteriol. 2000;182(13):3693–704.
10. Kimes NE, Grim CJ, Johnson WR, Hasan NA, Tall BD, Kothary MH, et al. Temperature regulation of virulence factors in the pathogen *Vibrio coralliilyticus*. ISME J. 2012;6(4):835–46.
11. Bina XR, Provenzano D, Nguyen N, Bina JE. *Vibrio cholerae* RND family efflux systems are required for antimicrobial resistance, optimal virulence factor production, and colonization of the infant mouse small intestine. Infect Immun. 2008;76(8):3595–605.
12. Bina JE, Provenzano D, Wang C, Bina XR, Mekalanos JJ. Characterization of the *Vibrio cholerae vexAB* and *vexCD* efflux systems. Arch Microbiol. 2006;186:171–81.
13. Hayashi M, Nakayama Y, Unemoto T. Recent progress in the Na^+-translocating NADH-quinone reductase from the marine *Vibrio alginolyticus*. Vol. 1505, Biochimica et Biophysica Acta. 2001. p. 37–44.
14. Matthey N, Drebes Dörr NC, Blokesch M. Long-Read-Based Genome Sequences of Pandemic and Environmental *Vibrio cholerae* Strains. Microbiol Resour Announc. 2018;7(23):1–3.
15. Fong JCN, Syed KA, Klose KE, Yildiz FH. Role of *Vibrio* polysaccharide (*vps*) genes in VPS production, biofilm formation and *Vibrio cholerae* pathogenesis. Microbiology. 2010;156(9):2757–69.
16. Ushijima B, Videau P, Poscablo D, Stengel JW, Beurmann S, Burger AH, et al. Mutation of the *toxR* or *mshA* genes from *Vibrio coralliilyticus* strain OCN014 reduces infection of the coral *Acropora cytherea*. Environ Microbiol. 2016;18(11):4055–67.
17. Ushijima B, Richards GP, Watson MA, Schubiger CB, Häse CC. Factors affecting infection of corals and larval oysters by *Vibrio coralliilyticus*. PLoS One. 2018;13(6):e0199475.
18. Skorupski K, Taylor RK. Control of the ToxR virulence regulon in *Vibrio cholerae* by environmental stimuli. Mol Microbiol. 1997;25(6):1003–9.
19. Ramos HC, Rumbo M, Sirard J-C. Bacterial flagellins: mediators of pathogenicity and host immune responses in mucosa. Trends Microbiol. 2004;12(11):509–17.
20. Liu R, Chen H, Zhang R, Zhou Z, Hou Z, Gao D, et al. Comparative transcriptome analysis of

Vibrio splendidus JZ6 reveals the mechanism of its pathogenicity at low temperatures. Appl Environ Microbiol. 2016;82(7):2050–61.
21. Burger AH. Quorum Sensing in the Hawai`ian Coral Pathogen *Vibrio coralliilyticus* strain OCN008. University of Hawaii at Manoa; 2017.
22. Hasegawa H, Häse CC. TetR-type transcriptional regulator VtpR functions as a global regulator in *Vibrio tubiashii*. Appl Environ Microbiol. 2009;75(24):7602–9.
23. Ball AS, Chaparian RR, van Kessel JC. Quorum Sensing Gene Regulation by LuxR/HapR Master Regulators in Vibrios. J Bacteriol. 2017;199(19):e00105-17.
24. Ben-Haim Y, Zicherman-Keren M, Rosenberg E. Temperature-regulated bleaching and lysis of the coral *Pocillopora damicornis* by the novel pathogen *Vibrio coralliilyticus*. Appl Environ Microbiol. 2003;69(7):4236–41.
25. Hasegawa H, Lind EJ, Boin MA, Häse CC. The extracellular metalloprotease of *Vibrio tubiashii* is a major virulence factor for pacific oyster (*Crassostrea gigas*) larvae. Appl Environ Microbiol. 2008;74(13):4101–10.
26. de O Santos E, Alves N, Dias GM, Mazotto AM, Vermelho A, Vora GJ, et al. Genomic and proteomic analyses of the coral pathogen *Vibrio coralliilyticus* reveal a diverse virulence repertoire. ISME J. 2011;5:1471–83.
27. Guillemette R, Ushijima B, Jalan M, Häse CC, Azam F. Insight into the resilience and susceptibility of marine bacteria to T6SS attack by *Vibrio cholerae* and *Vibrio coralliilyticus*. PLoS One. 2020;15(1):e0227864.
28. Winn KM, Bourne DG, Mitchell JG. *Vibrio coralliilyticus* Search Patterns across an Oxygen Gradient. PLoS One. 2013;8(7):1–8.
29. Sultan SZ, Silva AJ, Benitez JA. The PhoB regulatory system modulates biofilm formation and stress response in El Tor biotype *Vibrio cholerae*. FEMS Microbiol Lett. 2010;302:22–31.
30. Pratt JT, McDonough EK, Camilli A. PhoB regulates motility, biofilms, and cyclic di-GMP in *Vibrio cholerae*. J Bacteriol. 2009;191(21):6632–42.
31. Pratt JT, Ismail AM, Camilli A. PhoB regulates both environmental and virulence gene expression in *Vibrio cholerae*. Mol Microbiol. 2010;77(6):1595–605.
32. Fong JCN, Yildiz FH. The *rbmBCDEF* gene cluster modulates development of rugose colony morphology and biofilm formation in *Vibrio cholerae*. J Bacteriol. 2007;189(6):2319–30.
33. Fong JCN, Karplus K, Schoolnik GK, Yildiz FH. Identification and characterization of RbmA, a novel protein required for the development of rugose colony morphology and biofilm structure in *Vibrio cholerae*. J Bacteriol. 2006 Feb 1;188(3):1049–59.
34. Benitez JA, Silva AJ. *Vibrio cholerae* hemagglutinin(HA)/protease: An extracellular metalloprotease with multiple pathogenic activities. Toxicon. 2016 Jun;115:55–62.
35. Binesse J, Delsert C, Saulnier D, Champomier-Vergès MC, Zagorec M, Munier-Lehmann H, et al. Metalloprotease Vsm is the major determinant of toxicity for extracellular products of *Vibrio splendidus*. Appl Environ Microbiol. 2008;74(23):7108–17.
36. Aguirre-Guzmán G, Ruíz HM, Ascencio F. A review of extracellular virulence product of *Vibrio* species important in diseases of cultivated shrimp. Aquac Res. 2004;35:1395–404.
37. Norqvist A, Norrman B, Wolf-Watz H. Identification and characterization of a zinc metalloprotease associated with invasion by the fish pathogen *Vibrio anguillarum*. Infect Immun. 1990;58(11):3731–6.
38. Sussman M, Willis BL, Victor S, Bourne DG. Coral pathogens identified for White Syndrome (WS) epizootics in the Indo-Pacific. PLoS One. 2008;3(6).
39. Delston RB, Kothary MH, Shangraw KA, Tall BD. Isolation and characterization of a zinc-containing metalloprotease expressed by *Vibrio tubiashii*. Can J Microbiol. 2003;49:525–9.
40. Booth BA, Boesman-Finkelstein M, Finkelstein RA. *Vibrio cholerae* soluble hemagglutinin/protease is a metalloenzyme. Infect Immun. 1983;42(2):639–44.
41. Hase C, Finkelstein RA. Bacterial extracellular zinc-containing metalloproteases. Microbiol Rev.

1993;57(4):823–37.
42. Pollock FJ, Wilson B, Johnson WR, Morris PJ, Willis BL, Bourne DG. Phylogeny of the coral pathogen *Vibrio coralliilyticus*. Environ Microbiol Rep. 2010;2(1):172–8.
43. Denkin SM, Nelson DR. Induction of protease activity in *Vibrio anguillarum* by gastrointestinal mucus. Appl Environ Microbiol. 1999;65(8):3555–60.
44. Narita S, Masui C, Suzuki T, Dohmae N, Akiyama Y. Protease homolog BepA (YfgC) promotes assembly and degradation of β-barrel membrane proteins in *Escherichia coli*. Proc Natl Acad Sci USA. 2013;110(38):E3612–21.
45. Almagro-Moreno S, Kim TK, Skorupski K, Taylor RK. Proteolysis of Virulence Regulator ToxR Is Associated with Entry of *Vibrio cholerae* into a Dormant State. PLOS Genet. 2015;11(4):e1005145.
46. Almagro-Moreno S, Root MZ, Taylor RK. Role of ToxS in the proteolytic cascade of virulence regulator ToxR in *Vibrio cholerae*. Mol Microbiol. 2015;98(5):963–76.
